# MT1-MMP directs force-producing proteolytic contacts that drive tumor cell invasion

**DOI:** 10.1038/s41467-019-12930-y

**Published:** 2019-10-25

**Authors:** Robin Ferrari, Gaëlle Martin, Oya Tagit, Alan Guichard, Alessandra Cambi, Raphaël Voituriez, Stéphane Vassilopoulos, Philippe Chavrier

**Affiliations:** 1 Sorbonne Université, Institut Curie, PSL Research University, CNRS, UMR 144, 26 rue d’Ulm, F-75005 Paris, France; 2 Institut Curie, PSL Research University, CNRS, UMR 144, 26 rue d’Ulm, F-75005 Paris, France; 3 Department of Tumor Immunology, Radboud Institute for Molecular Life Sciences, Radboud University Medical Center, Nijmegen and Oncode Institute, Nijmegen, The Netherlands; 4 Department of Cell Biology, Radboud Institute for Molecular Life Sciences, Radboud University Medical Center, Nijmegen, The Netherlands; 5 Sorbonne Université, CNRS, UMR 8237, Jean Perrin Laboratory, Paris, France; 6 Sorbonne Université, INSERM UMRS 974, Institute of Myology, Paris, France

**Keywords:** Cancer, Breast cancer, Cell biology, Cell invasion, Invadopodia

## Abstract

Unraveling the mechanisms that govern the formation and function of invadopodia is essential towards the prevention of cancer spread. Here, we characterize the ultrastructural organization, dynamics and mechanical properties of collagenotytic invadopodia forming at the interface between breast cancer cells and a physiologic fibrillary type I collagen matrix. Our study highlights an uncovered role for MT1-MMP in directing invadopodia assembly independent of its proteolytic activity. Electron microscopy analysis reveals a polymerized Arp2/3 actin network at the concave side of the curved invadopodia in association with the collagen fibers. Actin polymerization is shown to produce pushing forces that repel the confining matrix fibers, and requires MT1-MMP matrix-degradative activity to widen the matrix pores and generate the invasive pathway. A theoretical model is proposed whereby pushing forces result from actin assembly and frictional forces in the actin meshwork due to the curved geometry of the matrix fibers that counterbalance resisting forces by the collagen fibers.

## Abstract (web summary)

The mechanism of force production by invadopodia is unclear. Here, the authors show that cell surface MT1-MMP when in contact with collagen, induces Arp2/3 branched actin polymerisation on the concave side of invadopodia, which generates a pushing force along with collagen cleavage by MT1-MMP to invade.

## Introduction

The migration of cells through tissues is essential during embryonic development, tissue repair, and immune surveillance^1^. Deregulated invasive migration is also key to disease processes, including cancer dissemination. Proteolytic degradation is indispensable for the tissue-penetrating properties of cancer cells, largely due to the high degree of intra- and intermolecular covalent cross-links found in type I collagen, the dominant extracellular matrix (ECM) found in native tissues, that consequently prevent the physical expansion of pre-existing pores to accommodate cell invasion^2–4^. It is believed that invasive cancer cells negotiate these tissue barriers by forming specialized F-actin-based protrusions, termed invadopodia, that focally degrade the ECM, thereby enabling cell penetration^5–7^. In this regard, MT1-MMP, a trans-membrane matrix metalloproteinase, is concentrated in invadopodia and is essential for the pericellular matrix degradation that marks carcinoma cell invasion across the basement membrane as well as dense, collagen-rich tissues^3,8–11^.

Although—by definition—all invadopodia types degrade the matrix and rely on MT1-MMP catalytic activity to do so, their structure and activity can differ depending on the composition and mechanical properties of the matrix environment^12–14^. In the classical model useful to study invadopodia formation, cancer cells are plated on a thin—quasi-two-dimensional (2D)—substratum of denatured collagen (i.e. gelatin) where degradative activity is concentrated in 0.5–1 μm diameter, actin-rich puncta^5^. In contrast, when exposed to more physiologic matrix construct of type I collagen fibers^4,9^, cancer cells assemble cortactin/F-actin-positive structures that further mature into degradation-competent invadopodia along with MT1-MMP focal delivery and accumulation in association with the underlying matrix of collagen fibers^14–17^. These structures, which elongate in the plane of the plasma membrane can typically be several microns in length^14,15,17^. We and others reported that cancer cells, which invade through the collagen gel with a nucleus-at-the-back configuration, preferentially form elongated invadopodia at the level of the advancing invasive protrusion ahead of the nucleus and degrade the matrix constricting fibers to support invasive path-generation^4,9,18^.

In addition to their ability to proteolytically remodel the ECM, tumor cells may also utilize cellular force to mechanically reorganize the ECM as they migrate through tissue barriers^19–21^. While it is known that invadopodia formation and activity can be regulated by matrix rigidity^22–25^, whether the invadopodial structures that form in association with collagen fibers are endowed with matrix-deforming activity that transmit forces to the surrounding ECM is presently unknown. Further, the mechanisms by which invasive cells coordinate topological and mechanical cues from the three-dimensional (3D) ECM environment with invadopodia organization and function during matrix degradation and invasion are largely unknown. As such, we set out to characterize the ultrastructural organization, dynamics, and mechanical properties of invadopodia generated at the interface between breast cancer cells and fibrillary type I collagen. Herein, using a combination of platinum replica electron microscopy, type I collagen fiber tracking and laser-induced rupture of collagen fibrils, we have uncovered a protease-independent role for MT1-MMP in controlling actin polymerization and mechanical force production at the invadopodial front. These data make a shift in the invadopodia paradigm wherein self-assembling, force-producing proteolytic cell–matrix contacts promote matrix pore enlargement to facilitate tumor-cell invasion.

## Results

### Bending and remodeling of collagen fibers by collagenolytic invadopodia

**Fig. 1:**
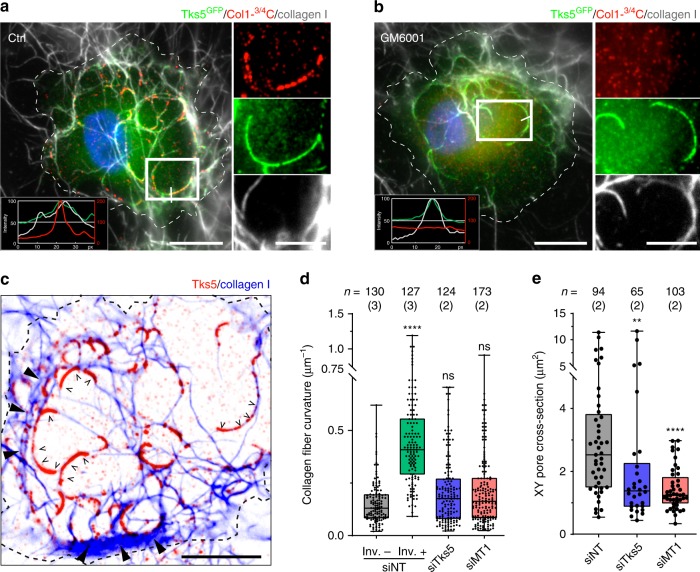
Collagen fiber bending and remodeling by matrix-degradative invadopodia. **a** MDA-MB-231 cells expressing Tks5^GFP^ (green) cultured on a thin collagen layer (gray) were stained for GFP (green), cleaved collagen neoepitope (Col1-¾C, red) and DAPI (blue). Fluorescence intensity profiles along the white line are showed in the inset. **b** GM6001-treated cells (40 µM) analyzed as in panel a. Scale bars, 10 µm; 5 µm (zoom-in). **c** Tks5 staining of MDA-MB-231 cells cultured on type I collagen for 60 min. Inverted lookup tables are used (collagen fibers in blue, Tks5-positive invadopodia in red). The cell contour is shown with a dashed line. Empty arrowheads point to curved invadopodia/fiber ensembles. Full arrowheads depict bundles of collagen fibers at the cell periphery. Scale bar, 10 µm. **d** Quantification of collagen fiber curvature (in µm^−1^) in association with Tks5-positive invadopodia (Inv. + ) or for invadopodia-free regions (Inv. -) underneath control (siNT-treated) cells, or in randomly selected regions of collagen fibers underneath siTks5- or siMT1-MMP-treated cells. Data are presented as box and whisker plots with black lines indicating medians and whiskers representing the 25th and 75th percentiles. *n*: number of fibers analyzed; (n): number of independent experiments. Statistical significance was determined by Kruskal–Wallis tests. **e** Box plots show the average pore cross sections between fibers quantified by confocal optical xy sections of fluorescently labeled rat tail collagen layer underneath the indicated cells. Medians and 25th and 75th percentiles are presented. *n*, number of cells analyzed; (*n*): number of independent experiments. Statistical significance was determined by Kruskal–Wallis tests. ** *P* < 0.01, **** *P* < 0.0001, ns not significant

Invasive MDA-MB-231 breast tumor cells seeded on a layer of fibrillar type I collagen formed curvilinear structures in association with the underlying fibers that were enriched in the invadopodial scaffolding protein, Tks5 (Fig. 1a)^14,18^. Staining with Col1-^3/4^C antibody that detects the collagenase-cleaved fragment of collagen I detected robust collagenolytic activity in association with Tks5^GFP^-positive structures, which was abolished upon treatment with the MMP inhibitor, GM6001 (Fig. 1a, b). Thus, Tks5-positive collagenolytic structures forming along collagen fibrils display the features of bona fide mature invadopodia^5–7^.

Conspicuous bending of collagen fibers in association with Tks5-positive invadopodia was visible (Fig. 1c). We used a circle-fitting method to estimate the curvature of the fibers underneath the ventral cell surface, and found that invadopodia-associated fiber segments were highly curved as compared to invadopodia-free regions of the fibers (Fig. 1d). In addition, we observed that collagen fibers were cleared underneath the cells (average pore size ~ 3.20 ± 0.37 μm^2^), and fibers were bundled at the cell edge (Fig. 1c, e).

**Fig. 2:**
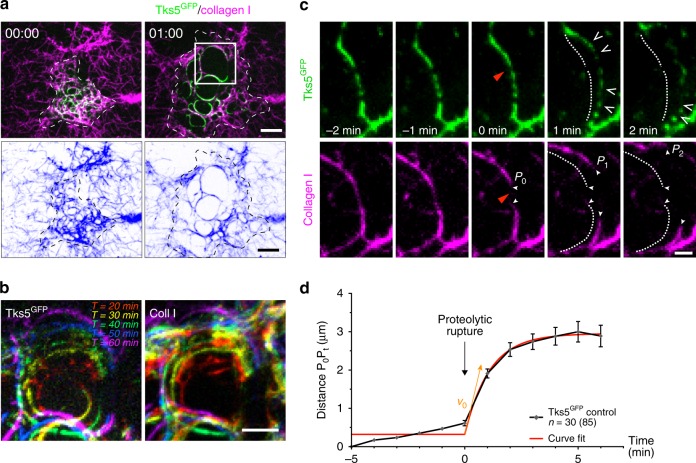
Force-production by Tks5-positive invadopodia. **a** MDA-MB-231 cells expressing Tks5^GFP^ (green) were plated on top of a thin layer of type I collagen (magenta) and imaged over time. Images represent the first and last frames from a representative movie (time in hr:min, see Supplementary Movie 1). The bottom row shows the collagen layer in an inverted lookup table (pseudocolored blue). Scale bar, 10 µm. **b** Color-coded time projections of five images made at 10-min intervals, corresponding to the boxed region in f, showing radial expansion of Tks5 invadopodia (upper image) and associated fiber (lower image) over time. Scale bar, 5 µm. **c** Gallery of consecutive frames from a time-lapse sequence of Tks5^GFP-^expressing cells (green) plated on a type I collagen layer (magenta). The gallery shows an invadopodia/collagen-fiber ensemble undergoing collagenolytic rupture at time 0 (red arrowhead, see Supplementary Movie 2). Rupture is followed by the elastic recoil of the invadopodia/collagen-fiber ensemble. The initial position of the invadopodia/collagen-fiber ensemble is shown by a dashed line and positions of the collagen fiber tips after rupture are indicated (lower row, white arrowheads). Open arrowheads point to regions of invadopodia disassembly (upper row). Time is indicated in min. Scale bar, 2 µm. **d** Invadopodia/collagen-fiber tension. The distance between the position of the collagen fiber tip (*P*_t_) and initial position (*P*_0_) was calculated and plotted over time. The black curve represents the mean from 85 proteolytic rupture events aligned at rupture time point (*t*_0_). Error bars, SEM; *n*: number of cells analyzed from three independent experiments. The curve shows typical viscoelastic recoil after proteolytic rupture and was fitted to a “plateau followed by one-phase association” model (red)

Live-cell imaging showed that Tks5^GFP^-positive invadopodia on the ventral cell surface were highly dynamic, with an average lifetime of ~ 41 ± 1.7 min (Supplementary Table 1) and that they elongated along the underlying collagen fiber at a rate of 0.15 ± 0.02 µm/min, producing typical bow- or ring-shaped structures (see Supplementary Movie 1, Fig. 2a, b, Supplementary Table 1 and Figure 1). Timed sequences demonstrated that invadopodia/collagen-fiber ensembles underwent homothetic expansion over time, with an average radial velocity of 0.16 ± 0.02 µm/min (Fig. 2b, Supplementary Table 1 and Supplementary Movie 1). Complementing this activity, we frequently observed proteolytic rupture and recoil movement of the invadopodia/collagen-fiber ensemble (Fig. 2c, Supplementary Table 1 and Supplementary Movie 2). The measurement of fiber relaxation as a function of time revealed a typical viscoelastic behavior of the invadopodia/fiber ensemble with an initial velocity *V*_0 = _3.1 ± 0.22 µm/min (Fig. 2c, d and Supplementary Table 1), which characterizes the tension-to-drag ratio of the fiber^26^. Overall, these observations suggest a strong remodeling capacity of collagenolytic invadopodia based on MMP activity, and demonstrate that the invadopodia-associated collagen fibers sustain mechanical tension and bending moment, which relax upon proteolytic rupture, demonstrating that cells produce and transmit force to the fibers at the level of invadopodia.

### Ultrastructural organization of actin filaments in collagenolytic invadopodia

**Fig. 3:**
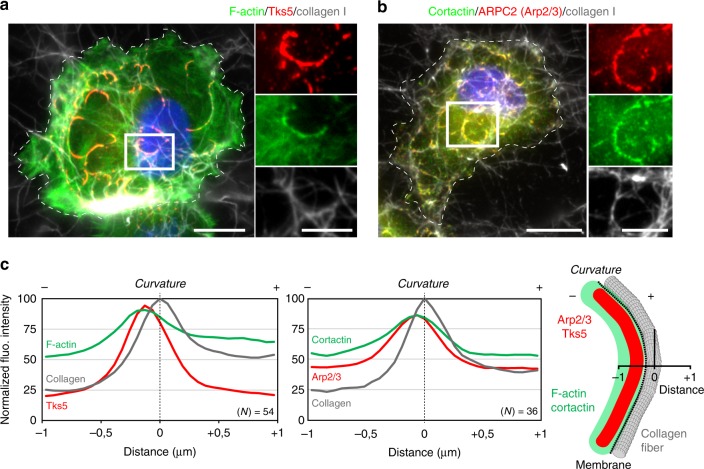
Invadopodia assemble on the inner side of the curved collagen fibrils. **a** Tks5 (red) and F-actin (green) staining of MDA-MB-231 cells cultured on type I collagen (gray) for 60 min (same image as in Fig. 1c). The nucleus is stained with DAPI (blue). The cell contour is shown with a dashed line. Insets show higher magnification of the boxed region in separated channels. **b** ARPC2 (Arp2/3 complex, red) and cortactin (green) staining as in panel a. Scale bars, 10 µm; 5 µm (zoom-in). Same image as in panel a using inverted lookup tables (collagen fibers in blue, Tks5-positive invadopodia in red). **c** Averaged fluorescence intensity profiles for the indicated markers. Individual line-scans were recorded perpendicular to the curved collagen fibers and aligned relative to the longitudinal fiber axis corresponding to the maximum intensity of collagen fluorescence set to 100 (position ‘0’). Distance in µm, (−) corresponds to the inner (concave) side of curvature, ( + ) correspond to the outer (convex) side of curvature. (*N*), number of individual line-scans that were averaged from respectively 32 and 20 cells from two independent experiments. The distribution of invadopodial components in association with a collagen fiber relative to curvature is sketched out

To gain insight into the processes underlying invadopodia-mediated force generation, we monitored the fluorescence intensity of several invadopodia components including F-actin, Tks5, the actin nucleating Arp2/3 complex and the F-actin binding protein, cortactin, along a line (i.e. line-scan) perpendicular to the collagen fiber and averaged for several invadopodia aligned relative to the longitudinal fiber axis (i.e. peak intensity of collagen fluorescence). We observed a striking polarization of the cytoskeletal actin and Tks5 components of invadopodial assemblies that predominantly accumulated on the inner (-) side of curved invadopodium/fiber ensembles, where 49 out of 54 invadopodia showed a peak of F-actin/Tks5 fluorescence on the inner side of the curved collagen fibril (Fig. 3a–c and Supplementary Table 1).

**Fig. 4:**
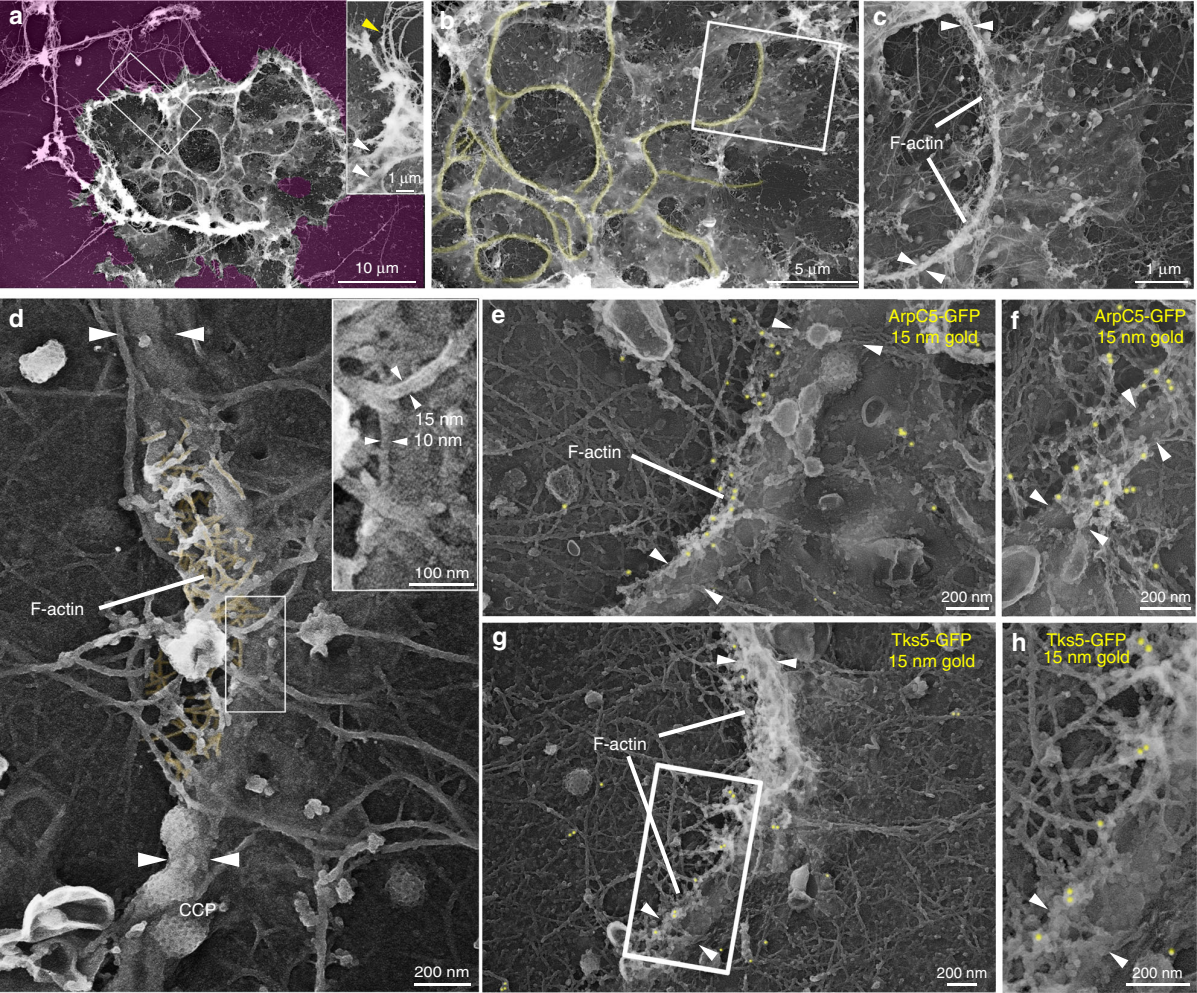
Ultrastructural organization of collagenolytic invadopodia. **a** Platinum replica electron microscopy (PREM) survey view of the cytoplasmic surface of the adherent plasma membrane in unroofed MDA-MB-231 cells plated for 60 min on a thin layer of collagen I (image is inverted). Extracellular space is pseudo-colored in purple. Inset is a zoom-in view of the boxed region to show the continuity of the collagen fibers outside (yellow arrowhead) and underneath the ventral plasma membrane (white arrowheads). Scale bars, 10 µm; 1 µm (inset). **b** PREM image showing increased electron density (pseudo-colored in yellow) corresponding to curvilinear collagen fibers underneath the ventral plasma membrane. Scale bar, 5 µm. **c** Zoomed-in region corresponding to the boxed region in panel **b**. Arrowheads indicate bow-shaped proteinaceous material in association with the membrane above a collagen fiber. Scale bar, 1 µm. **d** High magnification PREM view of a bow-shaped collagen fiber (arrowheads) and associated branched actin network (pseudo-colored in yellow) along the concave side of the fiber, i.e. an invadopodium. Clathrin-coated pits (CCP), some pinching the underlying collagen fibers, are visible. Inset is a zoomed-in view of the boxed region showing the differential width of ~ 10 nm-wide actin filaments and ~ 15 nm wide intermediate filament. Scale bars, 200 nm; 100 nm (inset). **e**, **f** Anti-GFP immunogold PREM of MDA-MB-231 cells expressing Arp2/3 complex subunit ArpC5B^GFP^. Immunogold beads are pseudo-colored in yellow. A zoomed-out PREM image corresponding to panel e is shown in Supplementary Figure 1c. Collagen fibers, arrowheads. Scale bars, 200 nm. **g**, **h** Anti-GFP immunogold PREM analysis of MDA-MB-231 cells expressing Tks5^GFP^. Panel h is a zoomed-in view of the boxed region in **g**. A zoomed-out PREM image corresponding to panel e is shown in Supplementary Figure 1f. Collagen fibers, arrowheads. Scale bars, 200 nm

We next used platinum replica electron microscopy (PREM) to define the cytoskeletal architecture of collagenolytic invadopodia at the ultrastructural level. Collagen fibers that could be tracked underneath the ventral plasma membrane because of their electron density (appear white in inverted PREM images), showed curvilinear trajectories confirming immunofluorescence imaging data (Fig. 4a–c and Supplementary Figure 2a,b). At higher magnification, actin filaments could be easily distinguished from other cytoskeletal elements based on their characteristic width (i.e. ~ 10 nm wide actin filaments vs. ~ 15 nm wide intermediate filaments, Fig. 4d, inset). Under these conditions, a network of branched actin filaments could be observed on the cytosolic face of the plasma membrane in association with the inner side of the curved collagen fibers (Fig. 4d). Accumulation of the Arp2/3 complex component, ARPC5, was detected by immunogold labeling in association with the branched actin filament network, representing an active actin nucleation complex at the inner plasma membrane interface with collagen fibers (Fig. 4e, f and Supplementary Figure 2c–e). Similarly, immunogold labeling of Tks5^GFP^ confirmed its enrichment at the inner rim of collagen fibers (Fig. 4g, f and Supplementary Figure 2f–h).

Highlighting the importance of Tks5 in invadopodia formation, siRNA-mediated knockdown of the protein significantly reduced F-actin-positive invadopodia formation and collagenolysis (Supplementary Figure 3a,b). Consistent with the observed decrease in invadopodia formation and activity, we found only low curvature of the collagen fibers in association with Tks5-silenced cells, similar to that observed in invadopodia-free fibers found in contact with normal cells (Fig. 1d and Supplementary Figure 3c). Concurrently, remodeling of collagen fibers was impaired in Tks5 knockdown cells, resulting in a ~ 2-fold reduction in matrix pore size underneath the cells (Fig. 1e and Supplementary Figure 3c). At the PREM level, collagen fibers found underneath the Tks5-silenced cancer cells were straightened, while branched actin assembly was abolished (Supplementary Figure 3d–g). These data identify Tks5 as a key component involved in the formation of collagenolytic invadopodia in MDA-MB-231 cells consistent with its strong pro-invasive and pro-metastatic potential^27^.

### MT1-MMP mediates invadopodia formation along collagen fibers

These observations revealed that invadopodia formed very selectively at plasma membrane/matrix contact sites, implying the activation of specific collagen receptor(s). Integrins and DDRs have been implicated in invadopodia formation, depending on matrix composition and organization, although with conflicting results^13,28,29^. We observed that silencing of β1 integrin or DDR1 collagen receptors in MDA-MB-231 cells had no effect on the assembly of Tks5-positive structures (Supplementary Figure 4a–d). However, DDR1 transcript levels were barely detectable in basal-like breast cancer MDA-MB-231 cells as previously reported^30,31^. Alternatively, we silenced DDR1 in mammary epithelial MCF10DCIS.com cells expressing ~ 60-fold higher level than mesenchymal MDA-MB-231 cells (Supplementary Figure 4e). Under these conditions, DDR1 knockdown resulted in a strong increase in Tks5 invadopodia assembly, thereby identifying its role as a repressor rather than an activator of invadopodia formation (Supplementary Figure 4f–h).

**Fig. 5:**
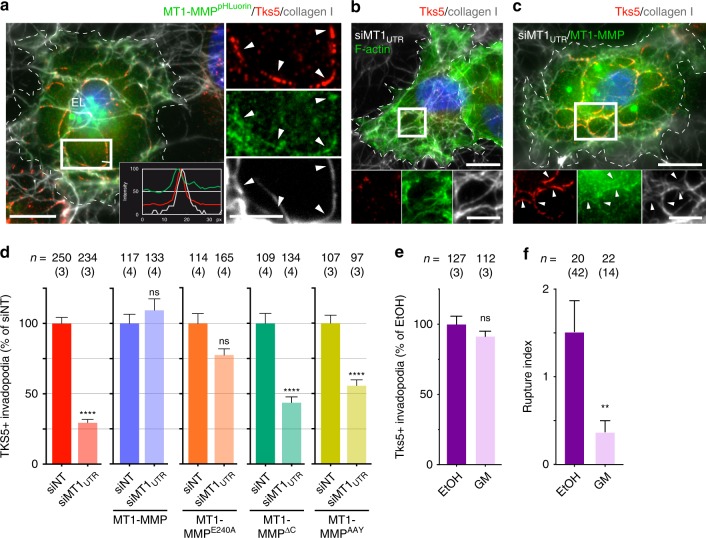
MT1-MMP is required for invadopodia assembly independently of its catalytic activity. **a** MDA-MB-231 cells expressing MT1-MMP^pHLuorin^ were cultured on type I collagen (gray), stained for GFP (green), Tks5 (red) and DAPI (blue). Right panels are zoom-in of the boxed region with separated channels. Arrowheads point to surface MT1-MMP^pHLuorin^ signal localized to a Tks5-positive invadopodium in association with a collagen fiber. Normalized fluorescence intensity profiles along the white line are shown in the inset. EL, perinuclear endolysosomes positive for MT1-MMP^pHLuorin^. Scale bar, 10 µm; 5 µm in zoom-in. **b** Cells were treated with siRNA against MT1-MMP 3′ and 5′ UTR sequences (siMT1_UTR_) and plated on collagen (gray) and stained for F-actin (green), Tks5 (red) and DAPI (blue). Bottom panels are zoom-in of the boxed region with separated channels. Scale bar, 10 µm; 5 µm in zoom-in. **c** Cells treated with siMT1_UTR_ siRNA as in panel b were transfected with MT1-MMP^pHLuorin^ rescue construct and stained for GFP to detect MT1-MMP^pHLuorin^ (green), Tks5 (red) and DAPI (blue). Bottom panels are zoom-in of the boxed region with separated channels. Arrowheads point to MT1-MMP^pHLuorin^ in Tks5-positive invadopodia. Scale bar, 10 µm; 5 µm in zoom-in. The cell contour is shown with a dashed line (panels a–c). **d** Quantification of Tks5-positive invadopodia in MDA-MB-231 cells knocked down for MT1-MMP or mock-treated and rescued with the indicated MT1-MMP constructs (see Supplementary Figure 5). The Y-axis is the ratio of the Tks5 area to the total cell area normalized to the mean value of corresponding Mock-treated cells (as percentage). *n*: number of cells analyzed; (*n*): number of independent experiments. Mann–Whitney tests. **e** Quantification of Tks5-positive invadopodia in MDA-MB-231 mock- (EtOH) or GM6001 (GM)-treated cells plated on type I collagen (see images in Supplementary Figure 5d,e) as in d. *n* number of cells analyzed from three independent experiments. Mann–Whitney test. **f** Rupture index (i.e. rupture events/cell/hr) calculated in mock- and GM-treated cells. Data are presented as the mean from three independent experiments; Mann–Whitney test. *n*: cell number; (*n*): invadopodia number. Error bars SEM, ** *P* < 0.01, **** *P* < 0.0001; ns not significant

Given these results, we shifted our attention to the fact that MT1-MMP has been reported to interact with type I collagen through both its catalytic and hemopexin ectodomains^32,33^. As expected, surface-exposed MT1-MMP accumulated in Tks5-positive invadopodia (Fig. 5a), and MT1-MMP knockdown abolished collagenolysis while impairing the bending and remodeling of the collagen fibers (Figure 1d, e and Supplementary Figure 3b). Strikingly, silencing of MT1-MMP resulted in a severe reduction of the assembly of mature Tks5-positive invadopodia (Fig. 5b, d and Supplementary Figure 5a). While our experimental setup based on Tks5 detection was unsuitable to identify Tks5-negative invadopodia precursors^7,34,35^, our data raise the possibility that MT1-MMP plays a direct regulatory role in the formation of mature matrix-degradative invadopodia. Indeed, assembly of Tks5-positive invadopodia in MT1-MMP depleted cells could be restored by the re-expression of wild-type MT1-MMP (Fig. 5c, d and Supplementary Figure 5a). Unexpectedly, however, invadopodia assembly could also be rescued by a catalytically inactive form of MT1-MMP with a point mutation in its active site (MT1-MMP^E240A^)^36^ (Fig. 5d and Supplementary Figure 5a–c). Similarly, inhibition of MT1-MMP catalytic activity with GM6001, although it potently suppressed collagen degradation and decreased the frequency of collagen rupture events, did not impair Tks5-positive invadopodia assembly (Figs 1b, 5e, f and Supplementary Figure 5d,e). Hence, while the assembly of Tks5-positive invadopodia in association with collagen fibers required surface-exposed MT1-MMP, its catalytic activity was dispensable. Interestingly, the cytosolic tail of MT1-MMP, in particular an associated LLY motif, have been shown to play important roles in controlling MT1-MMP trafficking and surface localization^37–40^. Hence, we assessed the ability of either a tail-deleted or LLY/AAY mutations to rescue invadopodia formation in MT1-MMP-silenced MDA-MB-231 cells. Interestingly, invadopodia rescue required the cytoplasmic tail of MT1-MMP, especially the integrity of the LLY motif (Figure 5d and Supplementary Figure 5a,b,f,g). Collectively, these data indicate that while surface expression of wild-type MT1-MMP is required for Tks5 recruitment and formation of mature, collagenolytic invadopodia, invadopodia assembly per se proceeds in the absence of its catalytic activity.

### MT1-MMP proteolytic activity contributes to invadopodia expansion and collagen remodeling

**Fig. 6:**
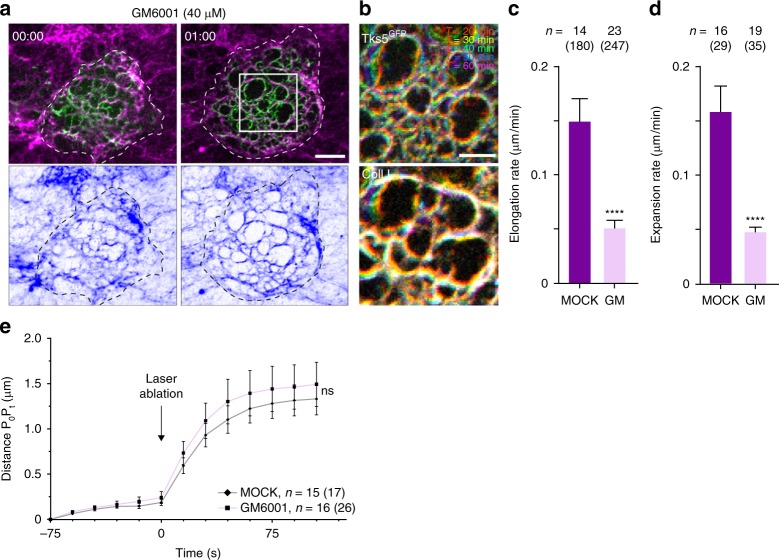
MT1-MMP proteolytic activity contributes to invadopodia expansion. **a** MDA-MB-231 cells expressing Tks5^GFP^ (green) were plated on top of a thin layer of type I collagen (magenta), treated with the MMP-inhibitor GM6001 and analyzed by live cell imaging as in Fig. 1f (see Supplementary Movie 3). **b** Color-coded time-projections of five images made at 10-min intervals, corresponding to the boxed region in **a**. **c** Elongation rate of invadopodia along collagen fibers in cells treated with GM6001 (GM) compared to mock treatment. Data are presented as the mean from three independent experiments; Mann–Whitney test. *n*: number of cells; (*n*): number of invadopodia. **d** Radial invadopodia expansion rate in cells treated with GM6001 compared to mock treatment. Data are presented as the mean from three independent experiments; Mann–Whitney test. *n*: cell number; (*n*): invadopodia number. **e** Displacement of an invadopodia/collagen-fiber ensemble before and after laser-ablation in mock- (gray) and GM-treated cells (pink) (see Supplementary Movie 4). *n*: cell number; (*n*): invadopodia number from three independent experiments. Data were analyzed using two-way ANOVA with Sidak’s multiple comparisons test for each time point. Error bars SEM, **** *P* < 0.0001, ns not significant

**Fig. 7:**
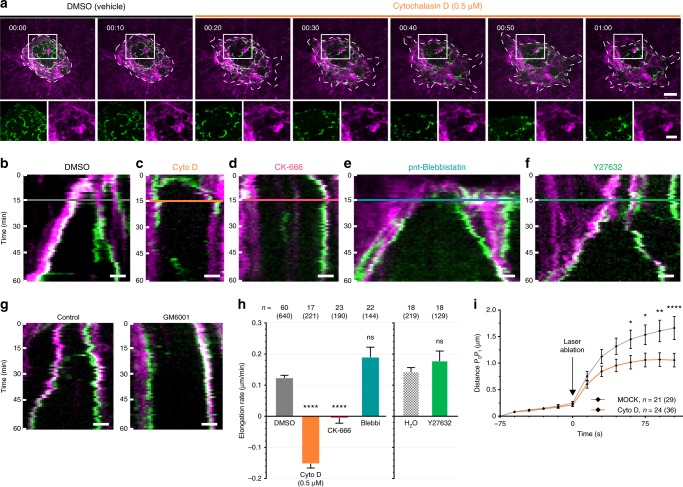
Invadopodial force production is powered by actin polymerization. **a** MDA-MB-231 cells expressing Tks5^GFP^ (green) were plated on a thin layer of type I collagen (magenta) and imaged over time. Cytochalasin D (0.5 µM) was added 15 min after starting the time-lapse. Representative frames (time in hr:min) show rapid disassembly following CytoD treatment and limited collagen-fiber remodeling (Supplementary Movie 5). The cell contour is shown with a dashed line. Scale bar, 10 µm. The lower row represents separate channels in the boxed region. Scale bar, 5 µm. **b**–**g** Kymograph analysis of radial expansion of an invadopodia/collagen-fiber ensemble upon drug addition (see Supplementary Movies 3 and 6 to 8). Drugs were added 15 min after starting the time-lapse (see colored lines), except in panel **g**, in which GM6001 was added at the beginning of the movie. Scale bars, 2 µm. **h** Quantification of the invadopodia elongation rate along collagen fibers in MDA-MB-231 cells treated with the indicated drugs. Data are presented as the mean from three independent experiments (DMSO, 8 experiments). n: cell number; (n): invadopodia number. Kruskal–Wallis and Mann–Whitney (Y27632 *vs*. H_2_0) tests. **i** Displacement of invadopodia/collagen-fiber ensemble over time before and after laser-ablation in low-dose CytoD (100 nM, orange) and mock-treated cells (gray). Curves represent the mean of 29 (Mock) and 36 (CytoD) curves aligned at rupture time-point (*t*_0_). *n*: number of cells analyzed from three independent experiments. Two-way ANOVA with Sidak’s multiple comparisons test for each time point. Error bars SEM, * *P* < 0.05, ** *P* < 0.01, **** *P* < 0.0001, ns not significant

Strikingly, longitudinal invadopodia growth and radial expansion of the invadopodia/matrix ensemble significantly slowed upon inhibition of MMP activity by GM6001 (Supplementary Movie 3, Fig. 6a, b and cd and Fig. 7g). In an effort to directly probe cell-mediated tension on collagen fibrils, we performed laser ablation of invadopodia/collagen-fiber ensembles in MDA-MB-231 cells cultured in the absence or the presence of GM6001. Importantly, displacement curves were similar under both conditions, with no significant difference in initial recoil velocity related to the tension-to-drag ratio (Fig. 6e, Supplementary Figure 6a–c, and Movie 4). Therefore, although there was no direct requirement for MT1-MMP catalytic activity in invadopodia assembly and in force production, proteolysis of type I collagen molecules appears essential for matrix pore dilation, possibly by either increasing fiber compliance or facilitating inter-fiber sliding.

### Actin polymerization-based force production at invadopodia

Actin-based mechanisms of force production can be mediated by myosin molecular motors or through the polymerization of actin filaments, which push the plasma membrane forward^41,42^. To characterize the mechanism of invadopodial force production, MDA-MB-231 cells were treated with cytochalasin D (CytoD, 0.5 µM), an inhibitor of actin polymerization^43^. This treatment strongly impaired longitudinal and radial invadopodial growth and triggered their rapid disassembly (Fig. 7a–c and h and Supplementary Movie 5). Similar effects were observed upon inhibiting the Arp2/3 actin nucleating complex with CK-666 (Fig. 7d, h, Supplementary Figure 7a,b and Movie 6)^44^. Of note, dilated pores found in the underlying matrix did not recoil to their initial size upon invadopodia disassembly induced by CytoD or CK666 treatment, indicating that forces applied by invadopodia to the matrix fibers induced permanent structural rearrangements in the collagen gel (Fig. 7c, d and Supplementary Movies 5 and 6). Unexpectedly, paranitro-blebbistatin, a non-phototoxic form of non-muscle myosin II inhibitor, blebbistatin, that ablates cell traction^45,46^, did not affect invadopodia elongation or radial expansion. Similarly, inhibition of myosin regulatory light chain phosphorylation by the p160ROCK inhibitor, Y-27632^47^, did not affect invadopodia dynamics (Figure 7e, f and h, Supplementary Figure 7c,d and Movies 7 and 8). Consistent with these results, non-muscle myosin II heavy chain (NMHC)-IIA does not associate with Tks5-positive invadopodia (Supplementary Figure 7e). In addition, myosin-II inhibition did not interfere with the bundling of the collagen fibers at the cell periphery consistent with the idea that pushing forces, rather than cell contractility and pulling forces, contribute to matrix clearance (Supplementary Figure 7c,d). Overall, these findings support a prominent role for an actin polymerization-dependent protrusive activity in supporting invadopodia pushing forces generation. This role was confirmed by laser ablation of collagen fibrils in cells treated with a lower dose of CytoD (100 nM) to reduce actin polymerization without triggering the rapid disassembly of pre-existing invadopodia. Under these conditions, while the initial recoil velocity was unperturbed after laser-induced collagen fiber rupture (Fig. 7i and Supplementary Figure 7f), the amplitude of invadopodia/collagen-fiber displacement was significantly reduced as shown by the lower plateau values (Fig. 7i). As stronger forces were applied by invadopodia under control conditions relative to CytoD-treated cells, we conclude that actin polymerization directly powers invadopodia-produced forces.

### Matrix pore opening during 3D invasion is driven by ring-like invadopodia expansion

**Fig. 8:**
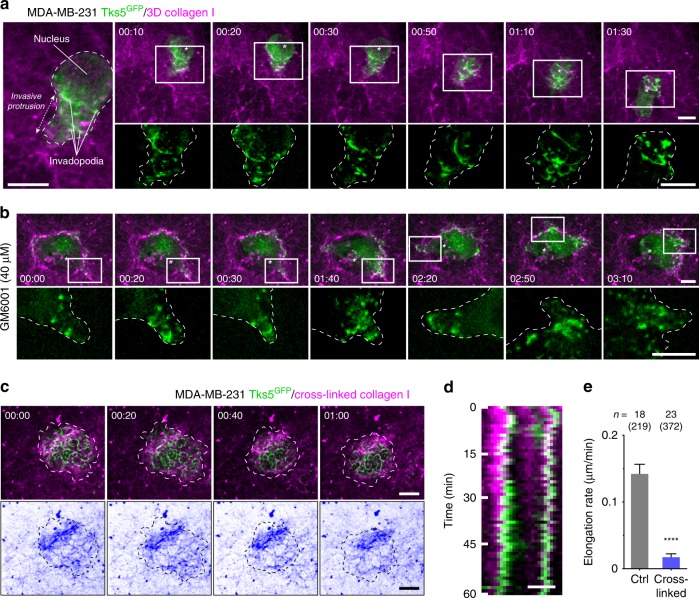
Force transmission and weakening of matrix counter-resistance by collagenolytic invadopodia. **a** Still image from a representative time-lapse sequence of Tks5^GFP-^expressing MDA-MB-231 cells (green) embedded in a 3D-collagen gel (magenta). The nucleus is located at the cell rear. Tks5-positive invadopodia form ahead of the nucleus at the level of the advancing invasive protrusion. The right panel shows a gallery of non-consecutive frames (time in h:min) from the time-lapse sequence (see Supplementary Movies 9–10). The bottom row shows a zoom-in of the boxed region for the GFP channel. Tks5^GFP^-positive structures form in association with constricting collagen fibers and expand in size during cell penetration. The cell contour is shown with a dashed line. * nucleus position based on the absence of a GFP signal. **b** Same as in **a** for GM6001-treated cells (time in hr:min, see Supplementary Movie 11). The expansion of Tks5^GFP^-positive structures forming in association with constricting collagen fibers (arrowheads) is reduced as compared to the control situation, which prevents cell transmigration into the ECM. The cell contour is shown with a dashed line. * nucleus position. Scale bars, 10 µm; 5 µm (zoom-in). **c** Tks5^GFP^-expressing cells were plated on cross-linked collagen (4% PFA, magenta) and analyzed by time-lapse microscopy. The gallery shows non-consecutive frames from a representative movie obtained from three independent experiments (time in h:min, see Supplementary Movie 12). The rigid collagen network is shown in the inverted images in the bottom row (pseudocolored blue). The cell contour is shown with a dashed line. Scale bars: 10 µm. **d** Kymograph analysis. Scale bar: 2 µm. **e** Elongation rate of invadopodia in cells plated on cross-linked collagen. Data are presented as the mean from three independent experiments. *n*: cell number; (*n*): invadopodia number. Mann–Whitney test. Error bars SEM, **** *P* < 0.0001

Invadopodia dynamics during confined migration in a 3D fibrous matrix environment has been largely overlooked. We monitored the invasion of MDA-MB-231 cells expressing Tks5^GFP^ in a 3D collagen gel (see Supplementary Movie 9). Tks5^GFP^-positive structures formed dynamically in association with constricting fibers at the level of the advancing invasive cell body, ahead of the bulky nuclear region (Fig. 8a, Supplementary Movie 10). We have previously reported that these Tks5-positive structures are *bona fide* collagenolytic invadopodia in 3D^18^. Some Tks5^GFP^ positive invadopodia appeared as ring-like structures that strapped the invasive protrusion and nuclear region like barrel hoops; some smaller Tks5^GFP^-invadopodia were also visible (Fig. 8a). Ring-like invadopodia expanded in size over time, with an average diameter growth rate of 0.09 ± 0.008 µm/min (see Supplementary Table 1). In sharp contrast, in the presence of GM6001 MMP inhibitor, cells could not invade through the 3D collagen gel and dynamic Tks5-rich assemblies were visible at the level of short cellular protrusions that formed in different regions of the cell periphery and quickly regressed as invasion was impaired (Fig. 8b and Supplementary Movie 11). These Tks5-positive structures showed a ~ 3-fold reduced growth rate (0.03 ± 0.006 µm/min) as compared to control invading cells (Supplementary Table 1). All together, these findings suggest that expansion of circumferential matrix-degradative invadopodia associated with constricting fibers contributes to widening matrix pores during 3D invasion of breast cancer cells.

### Physical modeling of invadopodial actin-based force production

Given the well-established ability of actin filament polymerization to generate protrusive forces^41,42,48^, we sought to develop a theoretical model that aimed at identifying the physical mechanism of force production in the invadopodia/collagen-fiber ensemble that was based on our experimental observations (see Supplementary Note 1). To equilibrate global forces on a polymerizing actin meshwork, the protrusive force must be balanced by a reaction force applied on the meshwork, i.e. a polymerizing branched actin meshwork must buttress itself against another structure in order to generate a forward protrusive force. In the classical example of lamellipodia of adherent cells, the protrusive force that pushes the plasma membrane is balanced by reaction forces due to adhesion to the ECM transmitted to the actin meshwork via focal adhesions. In the case of invadopodia, countering actin is polymerized along collagen fibers in the absence of a solid substrate to provide reaction forces, so that a classical model cannot directly apply here. Alternatively, in our model, protrusion forces are generated in invadopodia/collagen-fiber ensembles in absence of solid substrate, but they are locally balanced by frictional forces that appear in the dynamic actin meshwork due to the curved geometry of the collagen fiber (see Eq. 1 and Supplementary Figure 8 in Supplementary Note 1). In other words, a growing filament pushes forward the collagen fiber, and pushes back on neighboring filaments, a possibility that can only hold for curved geometries. Of note, while this mechanism assumes for the sake of simplicity a viscous description of the actin meshwork, which is classically adopted at long enough time scales (seconds to minutes)^49^, it would also qualitatively apply for an elastic description of the meshwork; in the latter case, protrusion forces would be balanced by elastic forces in the meshwork that appear in curved geometry. We do not rule out that other, non-antagonistic, mechanisms could also contribute to the reaction force such as the elastic cortical actin meshwork underlying the expanding invadopodial actin network or even some level of friction of the invadopodial F-actin meshwork against the plasma membrane bilayer.

Matrix fiber elasticity opposes this force and represents the energetic cost to further bend and displace the ECM fiber (see Eq. 2 in Supplementary Note 1). Given orders of magnitude inferred from the literature and our observations (typically, the diameter and persistence length of the ECM fiber, actin gel viscosity, and the length of the contact), this model predicts that for any sufficient initial curvature of the collagen fiber, actin polymerization-based forces can trigger further deformation and radial expansion (see Eqs. 3 and 4 and Supplementary Figure 9 in Supplementary Note 1). Based on this model, forces required to remodel less compliant ECM fibers should scale up; with available orders of magnitudes, it is expected that a significant increase (typically more than 10-fold) in collagen fiber bending modulus should impair the bending induced by polymerization forces (see Supplementary Figure 9 in Supplementary Note 1).

Given this model, we assessed the effect of increasing collagen I gel stiffness on invadopodia expansion as a measure of force. While chemical cross-linking (4% paraformaldehyde^13^) drastically increased fiber resistance to deformation (as shown by atomic force microscopy, the elastic modulus of the cross-linked collagen I matrix increased ~40-fold, see Supplementary Figure 7g and Methods section), invadopodia still formed in association with cross-linked collagen fibers and collagenolysis occurred (Fig. 8c and Supplementary Figure 7h). However, matrix clearance underneath the ventral cell surface was impaired, as well as invadopodia elongation and radial expansion rates, which substantially decreased (Figure 8d, e, Supplementary Table 1 and Supplementary Movie 12). As predicted, these data show that increased matrix rigidity and possibly reduced fiber slippage, due to matrix cross-linking, resist invadopodia-based pushing forces.

## Discussion

Invadopodia are hallmarks of invasive cells that localize MT1-MMP activity to cell–matrix contact sites and allow tissue barrier penetration as a function of their matrix-degradative capacity. The classical model of invadopodia that combines small actin-driven plasma membrane protrusions with MMP activity is based mostly on the analysis of cancer cells that are plated on a gelatin monolayer as a substrate. Although the gelatin model has been powerful to identify several invadopodia components and define their function, it suffers from limitations, including the extreme rigidity of the coated substratum and the two-dimensionality of the glass-coated surface. As such, this model system does not recapitulate the early phases of a tissue-invasive program. As opposed to the classical model, we propose an alternative invadopodia paradigm wherein self-assembling, force-producing, proteolytic cell–matrix contacts are generated as a means of promoting ECM invasion. Our data support a mechanism in which surface-exposed MT1-MMP, independently of its collagenolytic activity, binds to the collagen fiber and initiates a signaling cascade leading to Tks5 recruitment and actin polymerization at plasma membrane/fiber contact sites. These findings complement and extend our previous work demonstrating that the polarized recycling of MT1-MMP from internal storage compartments to the cell surface ensures the local accumulation of MT1-MMP to generate fully mature, degradation-competent invadopodia, thereby promoting tissue-invasive activity^15,17,18,50^.

**Fig. 9:**
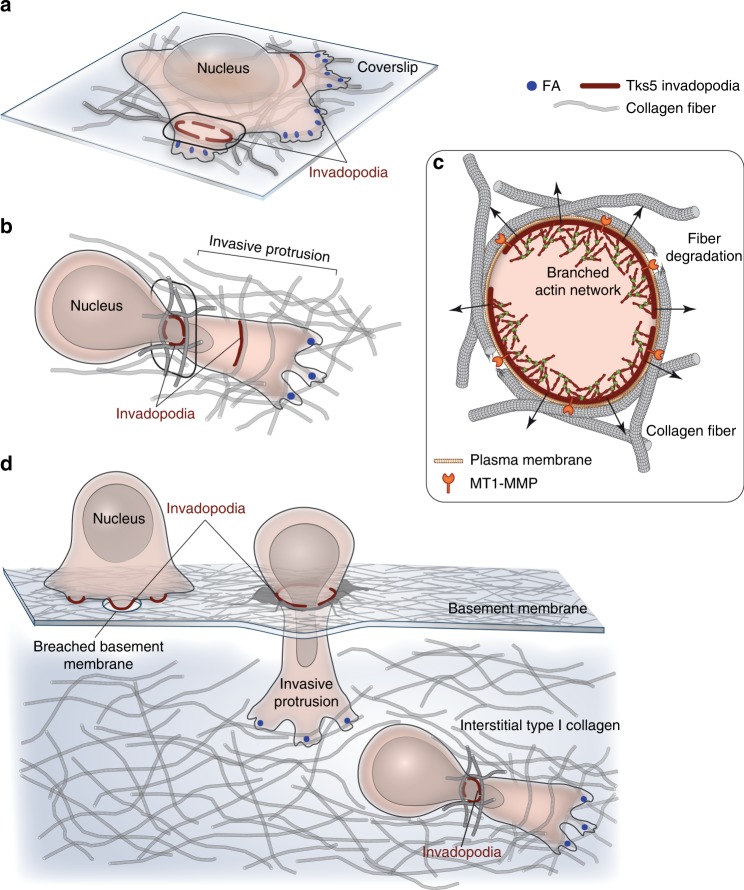
Model of actin-based proteolytic contacts during the early phases of the tissue-invasive cascade. **a** Tumor cells plated on top of a layer of fibrillar type I collagen form semi- to fully circular proteolytic contacts along collagen fibrils upon recognition of the collagen fibers by surface-exposed MT1-MMP, which correspond to invadopodia. Arp2/3 complex-mediated invadopodial actin assembly generates pushing forces against the collagen fibers, resulting in displacement of invadopodia-associated collagen fibers and matrix pore opening underneath the cell body. Due to the limited thickness of the collagen layer in this experimental setup (5–10 µm), cell invasion is impaired. **b** Confined migration of tumor cells through the dense 3D collagen network triggers invadopodia formation along collagen fibers, which constrict the nucleus^9,18^. MT1-MMP-mediated collagenolysis together with actin-driven force production cause matrix pore enlargement to promote nuclear transmigration and cell invasion. **c** Sketch of the physical model representing the invadopodial actin filament meshwork pushing on the collagen fiber (see Supplementary Figure 8 in Supplementary Note 1). Due to the curvature of the invadopodia/collagen-fiber ensemble, actin filaments are polymerizing and growing against their neighbors, therefore generating a force directed toward the collagen fiber that can further push, deform and displace the constricting fiber. This is potentiated by MT1-MMP proteolytic activity which lower collagen fiber bending energy and counter-resistance to this force. **d** We propose the following model of the early phases of the tissue-invasive cascade of carcinoma cells. Tumor invasion starts with the transmigration of the carcinoma cell through the basement membrane that involves MT1-MMP-mediated matrix proteolysis^10,11^. Small individual invadopodia producing focal basement membrane degradation may coalesce into a single ring-like structure at the interface between the cell and the quasi-2D basement membrane. Actin-based force generation at the ring-like invadopodia structure may help tear the basement membrane apart and widen the transmigration pore, which is then used by the cancer cell to project the invasive protrusion. In carcinomas, some emerging collective behaviors may induce a large area of basement membrane degradation (not drawn for simplicity). Once tumor cells reach the type I collagen-rich interstitial environment, proteolytic contacts form and promote cell invasion

Cells of the immune system, such as macrophages, also migrate through tissues thanks to F-actin-rich micrometer-sized invadopodia-like structures, termed alternatively as podosomes^5^. In recent studies, the protrusive force generated by macrophage podosomes was measured by visualizing nanoscale deformations of a synthetic, nanometer-thick, formvar-deformable membrane by atomic force microscopy. This elegant experimental design allowed the authors to propose a model whereby protrusive force at podosomes derives from actin assembly within the podosome actin core in combination with the contractility of radial actomyosin filaments that connect the actin core to a surrounding adhesive ring anchored to the substratum through integrins^51,52^. In the case of cancer cells plated on a gelatin substratum, punctate invadopodia were predominantly observed underneath the nucleus where nuclear indentations were detected at the interface between the invadopodia core and the juxtaposed nucleus^53^. Based on these observations, the authors proposed that the invadopodial actin meshwork could push against—and indent—the edge of the nucleus; based on considerations of force balance, these invadopodia would then be predicted to produce a protrusive force that could be transmitted to the ECM^53^. However, a direct contribution of the nucleus to force balance at the level of collagenolytic invadopodia of tumor cells migrating in a 3D fibrous matrix environment is questionable as invadopodia can form along and repel collagen fibers located at micrometer distances from the nucleus (see Figures 1a, b and 8a, and sketched out in Fig. 9a, b). Rather, we propose a model whereby, the energy of actin polymerization at the level of each individual invadopodium—independent of actomyosin—is efficiently transformed into an outward pointing force that can repel the confining matrix fibers while opening matrix pores via MT1-MMP-associated proteolytic activity. This force stems from the fact that a reaction force is generated by each polymerizing actin filament on its neighbors due to the curved geometry imposed by the curvature of the invadopodia/fiber ensemble (see Fig. 9c and Supplementary Note 1). A pre-requirement of this theoretical model is the curved geometry of the underlying fiber scaffold. Future work will be necessary to determine whether the preexisting curvature of native collagen fibers is sufficient to trigger branched actin-based force production or whether additional steps of collagen bending occur prior to invadopodia assembly. Along this line, some pushing forces produced by Tks5-positive protrusions at the level of the advancing cell body^19,20^ may trigger initial bending of constricting collagen fibers and favor invadopodia assembly along the curved fibers for subsequent fiber cleavage, repelling and bundling (Fig. 9b). Our data support the conclusion that pushing forces produced by invadopodia, acting cooperatively with proteolytic cleavage by MT1-MMP to increase matrix compliance, generate the invasive pathway.

In our model, MT1-MMP acts both as an initiator and executor component of invadopodia-linked invasion of type I collagen-rich barriers. However, this matrix-repelling, proteolytic contact model may also be relevant in the context of tumor cells traversing the BM where matrix pore could be enlarged after the initial proteolytic breach during the in situ-to-invasive carcinoma switch as well as during the intravasation or extravasation of blood vessels and lymphatics^8,10,54–56^. Along this line, recent findings suggest that the relevance of force-producing ECM-remodeling contacts may be generalized to non-invasive developmental BM breaching programs, such as early vulval development in *Caenorhabditis elegans*^57,58^. However, in the context of mammalian tissues, the ability of mechanical force alone to allow cancer cells to breach intact BM remains unclear. Alternatively, individual invadopodia structures may initially form as small proteolytic puncta that pierce the BM, in a process that precedes their clustering into a large ring, which could then widen the BM transmigration pore at the basis of the invasive protrusion of the carcinoma cell to facilitate transmigration (Fig. 9d). In any case, the ability of MT1-MMP to regulate both the mechanical and proteolytic activities of cancer cell invadopodia support a model wherein “cut and push” are purposefully linked to promote tissue-invasion programs.

## Methods

### Plasmid constructs

Construct expressing Tks5^GFP^ was a kind gift of Dr S. Courtneidge (OHSU, Portland, OR). Plasmid expressing GFP-ArpC5B was a kind gift of Dr. A. Gautreau (Ecole Polytechnique, Paris, FR). MT1-MMP with internal pHLuorin has been previously described^59^. E240A, ΔCter and LLY/A mutations were generated by PCR mutagenesis method (see Supplementary Figure 4b). MT1-MMP_LLY/A (forward primer: 5′-GGGACCCCCAGGCGAgccgccTACTGCCAGCGTTCC-3′), ΔCter (forward primer: 5′-GACGCCATGGGTGACCCAGGCGA-3′, E240A (forward primer: 5′-GTGGCTGTGCACGCGCTGGGCCATGCC-3′).

### Cell culture, stable and transient transfection, and siRNA treatment

MDA-MB-231 cells obtained from ATCC (ATCC HTB-26) were grown in L15 medium supplemented with 15% fetal calf serum and 2 mM glutamine at 37 °C in 1% CO_2_ and MCF10DCIS.com cell line was purchased from Asterand and maintained in DMEM- F12 medium supplemented with 2 mM glutamine and with 5% horse serum^60^. Cell lines were routinely tested for mycoplasma contamination. MDA-MB-231 cells stably expressing Tks5^GFP^ were generated by lentiviral transduction. For transient expression, MDA-MB-231 cells were transfected with plasmid constructs using Lipofectamine 3000 according to the manufacturer instructions (ThermoFisher). Cells were analyzed by live cell imaging 24–48 h after transfection. For knockdown, MDA-MB-231 and MCF10DCIS.com cells were treated with the indicated siRNA (50 nM) using Lullaby (OZ Biosciences, France) and analyzed after 72 h of treatment. siRNAs used for this study are listed in Supplementary Table 2.

### Antibodies and reagents

The source and working dilution of commercial antibodies and chemical reagents used for this study are listed in Supplementary Tables 3 and 4, respectively. Hepatocyte growth factor (HGF) was purchased from PeproTech Inc. and used at 20 ng/ml.

### Western blot analysis

Cells were lysed in SDS sample buffer, separated by SDS-PAGE, and detected by immunoblotting analysis with the indicated antibodies. Antibodies were visualized using the ECL detection system (GE Healthcare).

### RNA extraction, cDNA synthesis, and qPCR analysis

Cells were pelleted by centrifugation and washed in PBS prior lysis and RNA extraction using RNeasy Mini Kit from Qiagen. cDNAs were produced from 1 µg of extracted RNA using High capacity DNA reverse transcription kit from Applied Biosystem and used for quantitative PCR using LightCycler 480 SYBR Green I Master from Roche Life Science. Each condition was realized in triplicate with the following controls: a sample of RNA without reverse transcriptase, a sample without RNA but with reverse transcriptase, a sample without both, as well as an internal control with a GAPDH qPCR primer. The following qPCR primers were used: for MT1-MMP 5′-GGATACCCAATGCCCATTGGCCA-3′ and 5′-CCATTGGGCATCCAGAAGAGAGC-3′ at 600 nM, for DDR1 5′-CAACCACAGCTTCTCCAGTGGCTA-3′ and 5′-GCATGTTGTTACAGTGGACCTGCATA-3′ at 500 nM, and for GAPDH 5′-AGCCACATCGCTCAGACAC-3′ and 5′-GCCCAATACGACCAAATCC-3′ at 500 nM. qPCR reaction was performed using LightCycler® 480 thermocycling machine. Briefly, samples were pre-incubated for 5 min at 95 °C before undergoing 45 cycles of amplification composed of 20 s at 95 °C, 15 s at 60 °C and 15 s at 72 °C. A final cycle of 5 s at 95 °C and 1 min at 70 °C was performed before extracting melting curves for analysis. For each sample average of Cycle Thresholds (CTs) were calculated and extreme values filtered out if standard deviations were above 1. Differences between mean CT values of each sample and mean CT values of GAPDH sample were calculated to obtain ΔCT. Differences between mean CT values of each sample and ΔCT were calculated for each sample and squared to obtain the relative mRNA level expression of each sample as compared to GAPDH control. Values were then normalized on siNT-treated control set to 100 percent.

### Indirect immunofluorescence analysis of cells plated on collagen

Coverslips were layered with 200 µl of ice cold 2.0 mg/ml acidic extracted collagen I solution (Corning) in 1x MEM mixed with 4% Alexa-Fluor 647-conjugated type I collagen. The collagen solution was adjusted to pH 7.5 using 0.34 N NaOH and Hepes was added to 25 µM final concentration. After 3 min of polymerization at 37 °C, the collagen layer was washed gently in PBS and cells in suspension were added for 60 to 90 min at 37 °C in 1% CO_2_ before fixation. Cells were pre-extracted with 0.1% Triton X-100 in 4% paraformaldehyde in PBS during 90 s and then fixed in 4% paraformaldehyde in PBS for 20 min and stained for immunofluorescence microscopy with indicated antibodies.

### Line-scan analysis of averaged fluorescence intensity profiles

Fluorescence intensity profiles of type I collagen and of indicated antibodies were obtained using the line-scan function (average intensity) of Metamorph software analyzing a region perpendicularly crossing the curved collagen fibers associated with invadopodia markers (depicted by a white line). Intensity profiles were normalized to each maximum and plotted along the region of interest (distance in pixels) to visualize the presence or absence of peaks of fluorescence intensity along collagen fibers. For Col1-^3/4^C neoepitope (collagen degradation), intensity profiles were presented as raw fluorescence intensity to appreciate the absence of signal in GM6001-treated cells.

For Fig. 1h, several intensity profiles of identical length were aligned relative to a reference point set as the longitudinal fiber axis corresponding to the maximum intensity of collagen fluorescence (position ‘0’). All the aligned fluorescence intensity profiles (Tks5, F-actin, ARPC2 and cortactin) were then averaged and compared to the averaged collagen intensity profile. Distances were measured in µm; negative distances (−) corresponded to the inner side of the collagen fiber curvature, while positive distances ( + ) corresponded to the outer side of the curvature.

### Analysis of collagen fiber curvature and pore cross-section

The ThreePointCircularROI plugin in Fiji software was used to fit a circle along a collagen fiber at a given position. The reciprocal of the radius (in µm) extracted from this circle was defined as the collagen fiber curvature. Collagen fibers were selected based on co-occurrence or not with Tks5 signal in control (siNT-treated cells) situation to measure fiber curvature associated or not with invadopodia. In siMT1-MMP and siTks5-treated samples, several collagen fibers were randomly chosen and curvature was measured as above.

For XY pore cross-section measurement, the collagen signal beneath the cell area was extracted and blurred through the Smooth function in Fiji software. After removing the smooth continuous background using the subtracting background function in Fiji, the collagen signal was inverted and a manual threshold of the inverted image was applied to only keep surfaces devoid of collagen. This was further analyze using the Analyze Particles function in Fiji to determine the average area (in µm²) of the “particles” (i.e. roughly corresponding to matrix pores in the thick collagen gel experimental setup).

### Transmission electron microscopy of unroofed cells

Adherent MDA-MB-231 cells plated for 45–60 min on glass coverslips coated with a thin layer of collagen were disrupted by sonication as described previously^61^. Briefly, coverslips where rinsed three times in Ringer + Ca^2+^ (155 mM NaCl, 3 mM KCl, 3 mM NaH_2_PO_4_, 5 mM HEPES, 10 mM glucose, 2 mM CaCl_2_, 1 mM MgCl_2_, pH 7.2), then briefly incubated with 0.5 mg/ml poly-L-lysine in Ringer-Ca^2+^, quickly rinsed in Ringer-Ca^2+^ (155 mM NaCl, 3 mM KCl, 3 mM NaH_2_PO_4_, 5 mM HEPES, 10 mM glucose, 3 mM EGTA, 5 mM MgCl_2_, pH 7.2) and unroofed by scanning the coverslip with rapid sonicator pulses in KHMgE buffer (70 mM KCl, 30 mM HEPES, 5 mM MgCl_2_, 3 mM EGTA, pH 7.2). Paraformaldehyde 2%/glutaraldehyde 2%-fixed cells were further sequentially treated with 0.5% OsO_4_ in KHMgE buffer, 1% tannic acid and 1% uranyl acetate prior to graded ethanol dehydration and Hexamethyldisilazane substitution (HMDS, Sigma-Aldrich). For immunogold labeling, 4% paraformaldehyde-fixed plasma membranes were washed and quenched using 0.1% NaBH_4_ in KHMgE buffer for 10 minutes before 30 min saturation with BSA in KHMgE buffer, 1 hr incubation with primary antibody and two consecutive, 20 min each, incubations with goat anti-rabbit or anti-mouse secondary antibodies conjugated to 15 nm gold and further post-fixed with 2% glutaraldehyde. Dried samples were then rotary-shadowed with 2 nm of platinum and 5–8 nm of carbon using an ACE600 high vacuum metal coater (Leica Microsystems). Platinum replicas were floated off the glass by 5% hydrofluoric acid, washed several times by floatation on distilled water, and picked up on 200 mesh formvar/carbon-coated EM grids. The grids were mounted in a eucentric side-entry goniometer stage of a transmission electron microscope operated at 80 kV (Philips, model CM120) and images were recorded with a Morada digital camera (Olympus). Images were processed in Adobe Photoshop to adjust brightness and contrast and presented in inverted contrast. For analyses of Tks5-depleted cells, cells were transfected with indicated siRNAs (50 nM, Dharmacon) using Lullaby (OZ Biosciences, France) 72 h prior to sample preparation.

### Quantification of pericellular collagenolysis

Cells treated with indicated siRNAs were trypsinized and resuspended (2.5 × 10^5^ cells/ml) in 200 µl of ice cold 2.0 mg/ml collagen I solution prepared as described above. 40 µl of the cell suspension in collagen was added on glass coverslip and collagen polymerization was induced for 30 min by incubation at 37 °C. L-15 complete medium was then added and cells embedded in collagen were incubated for 16 h at 37 °C in 1% CO_2_. After fixation for 30 min at 37 °C in 4% paraformaldehyde in PBS, samples were incubated with anti-Col1-^3/4^C antibodies for 2 h at 4 °C. After extensive washes, samples were counterstained with Cy3-conjugated anti-rabbit IgG antibodies, Phalloidin-Alexa488 to visualize cell shape and mounted in DAPI. Image acquisition was performed with an A1R Nikon confocal microscope with a 40X NA 1.3 oil objective using high 455 sensitivity GaASP PMT detector and a 595 ± 50 nm band-pass filter. Quantification of degradation spots was performed as described^15^. Briefly, maximal projection of 10 optical sections with 2 µm interval from confocal microscope z-stacks (20 μm depth) were preprocessed by a Laplacian-of-Gaussian filter using a homemade ImageJ macro^15^. Detected spots were then counted and saved for visual verification. No manual correction was done. Degradation index was the number of degradation spots divided by the number of cells present in the field, normalized to the degradation index of control cells set to 100.

### Invadopodia assay

5 × 10^4^ cells were plated on collagen-coated coverslips, fixed after 60 min and stained with Tks5 and cortactin antibodies. Images were acquired with a wide-field microscope (Eclipse 90i Upright; Nikon) using a 100 × Plan Apo VC 1.4 oil objective and a highly sensitive cooled interlined charge-coupled device (CCD) camera (CoolSnap HQ2; Roper Scientific). A z-dimension series of images was taken every 0.2 µm by means of a piezoelectric motor (Physik Instrumente). For quantification of Tks5 associated with invadopodia, three consecutive z-planes corresponding to the plasma membrane in contact with collagen fibers were projected using maximal intensity projection in Fiji and Tks5 signal was determined using the thresholding command excluding regions < 8 px to avoid non-invadopodial structures. Surface covered by Tks5 signal was normalized to the total cell surface and values normalized to that of control cells.

### Live-cell imaging on type I collagen layer

For live imaging of cells on a fibrous collagen layer, glass bottom dishes (MatTek Corporation) were layered with 15 µl of a collagen solution as described above to produce a 5–10 µm thick layer of collagen. To cross-link collagen, polymerized collagen was incubated 20 min in PBS with 4% PFA and 5% Sucrose, and washed extensively in PBS before adding cell suspension in normal L-15 medium. 1 ml of cell suspension (7.5 × 10^4^ cells/ml) in complete medium was added and incubated for 30 min at 37 °C, 1% CO_2_. Z-stacks (11 images, 0.5 µm z-step) images were acquired every min during 60–90 min by confocal spinning-disk microscopy. For drugs treatment, cells were cultured in 1 ml of complete medium with vehicle (DMSO) and imaged every min for 15 min. Then, 1 ml of drug-containing medium (2x concentration) was added and cells were further imaged for 45 min. Image sequences were acquired on a spinning-disk (Roper Scientific) using a CSU22 Yokogawa head mounted on the lateral port of an inverted TE-2000U Nikon microscope equipped with a 40 × 1.4NA Plan-Apo objective lens and a dual-output laser launch, which included 491 nm and 561 nm, 50 mW DPSS lasers (Roper Scientific). Images were collected with a CoolSNAP HQ^2^ CCD camera (Roper Scientific). The system was steered by Metamorph 7 software. Kymographs were obtained with Fiji software along a line spanning the invadopodia diameter.

### Live-cell imaging in 3D type I collagen

Glass bottom dishes (MatTek Corporation) were layered with 10 µl of a solution of 5 mg/ml unlabeled type I collagen mixed with 1/25 volume of Alexa-Fluor 647-labeled collagen. Polymerization was induced at 37 °C for 3 min as described above and the bottom collagen layer was washed gently in PBS; 1 ml of cell suspension (1 × 10^5^ cells/ml) in complete medium was added and incubated for 30 min at 37 °C. Medium was gently removed and two drops of a mix of Alexa-Fluor 647-labeled type I collagen/unlabeled type I collagen at 2.0 mg/ml final concentration were added on top of the cells (top layer). After polymerization at 37 °C for 90 min as described above, 2 ml of medium containing 20 ng/ml HGF was added to the culture. Polymerized collagen gels were 100–150 µm thick based on optical sectioning by confocal spinning-disk microscopy and cells’ thickness was ~ 15–20 µm (see Supplementary Movie 9). Z-stacks of images were acquired every 10 min during 16 hrs by confocal spinning-disk microscopy as described above.

### Invadopodia elongation rate measurement

The length of Tks5-positive invadopodia, defined as curvilinear GFP-positive structures in association with collagen fibers, was analyzed overtime in cells plated on Alexa-Fluor 647-labeled collagen. Structures smaller than 10 px (~ 2 µm) were not taken into account in the analysis. Invadopodia elongation rate was calculated by dividing the increment length between initial and final time-points by the time interval (in µm/min). Positive growth rate corresponds to an increase of invadopodia length overtime (i.e. elongation), while negative growth rate represents a decrease of invadopodia length overtime (i.e. disassembly). In case of drug treatment, elongation rate was measured after drug addition.

### Time projections, kymographs and invadopodia radial expansion rate measurement

For visualization and quantification of invadopodia radial expansion rate, time projections and kymographs of expanding circular invadopodia were performed. We used the temporal color-code function in Fiji to assign a different color for each of the five frames with a 10-min interval from a time-lapse sequence recorded every min. In addition, for each circular invadopodia, a line spanning the invadopodia was drawn and a kymograph was extracted using Multi-kymograph function in Fiji. Radial growth rate of expanding invadopodia was calculated as followed: (Diameter *t*_n_-Diameter *t*_0_)/(*t*_n_-*t*_0_) (see Fig. 5b–g).

### Laser ablation and initial recoil velocity calculation

The laser ablation system was composed of a pulsed 355-nm ultraviolet laser (Roper Scientific) interfaced with an iLas system running in parallel with Metamorph 7 Software. This system was mounted on a confocal spinning disk (Yokagawa CSU-X1 spinning head on a Nikon Eclipse T*i* inverted microscope) equipped with an EM-CCD camera (Evolve, Photometrics) and a 100x oil immersion objective (Nikon S Fluor 100 × 0.5–1.3 NA). MDA-MB-231 cells expressing Tks5^GFP^ were plated on glass coverslips coated with a thin layer of Cy5-labeled collagen for 30 min at 37 °C. To allow acute ablation of a single invadopodia/collagen fiber ensemble, curvilinear invadopodia of a total length greater than 4.5 µm were selected. The ablation region was drawn as a line of 10–20 px long and 1 px thick crossing the middle of the invadopodia arc perpendicularly. Z-stacks (4 images, 0.5 µm z-step) of images were acquired at 15 s interval during 2 min before ablation. For photo-ablation, the laser beam was focused on the region of interest during a 10–20 ms pulse at 65–85% laser power. Laser ablation settings were validated by the absence of recovery of GFP and Cy5 signal recovery overtime (in contrast to FRAP). Z-stacks were acquired as above for 15 s interval during 3 min and then prolonged to 30 s interval for another 3 min to ensure full capture of the movement. Displacement of the invadopodia/collagen fiber ensemble from its position at *t*_0_ (rupture time), was calculated and the speed of fiber retraction at *t*_0_, i.e. “initial recoil velocity” (*V*_0_) was obtained after fitting the displacement curve with plateau followed by one-phase association exponential using GraphPad Prism (GraphPad Software)^26^.

### Atomic force microscopy (AFM) analysis and quantification of collagen stiffness

The local stiffness of collagen gels was measured with a Catalyst BioScope (Bruker, Germany) atomic force microscope coupled to a confocal microscope (TCS SP5II, Leica) using the “point and shoot” feature of the Nanoscope software (Bruker). Silicon nitride cantilevers with nominal spring constants of 0.7 N/m (Scanasyst-Fluid, Bruker) were used without any tip modification. The system was calibrated first in air and then in PBS prior to each experiment by measuring the deflection sensitivity on a glass surface, which enabled determination of the cantilever spring constant using the thermal noise method^62^. AFM height images captured in the PeakForce Tapping mode allowed for the selection of ‘point of interest’ to obtain the force curves. The forward (approach) and reverse (retraction) velocities were kept constant at 1 μm/s, ramping the cantilever by 0.5 μm with a 0.2 V (3.2 nN) threshold in a closed z loop. After baseline correction, approach curves were analyzed for determination of Young’s modulus of elasticity using Sneddon’s conical indenter model, for which Poisson’s ratio was set as 0.5 and the half angle of the indenter as 18°. Contact point-independent linear Sneddon equation was used for fitting the approach curves^63^. The region on the approach curve, through which the model was fit was determined via setting the lower and upper boundaries that corresponded to approximately 10 and 70% of the difference between the maximum and minimum forces exerted, respectively.

### Statistics and reproducibility

All results were presented as mean ± standard error of the mean (SEM) of three independent experiments. GraphPad Prism (GraphPad Software) was used for statistical analysis. Statistical significance was defined as *P < 0.05, **P < 0.01, ***P < 0.001, ****P < 0.0001, ns, not significant. Data were tested for normal distribution using the D’Agostino–Pearson normality test and nonparametric tests were applied otherwise. One-way ANOVA, Kruskal–Wallis, Mann–whitney or two-way ANOVA with Sidak’s test for multiple comparisons tests were applied as indicated in the figure legends. All experimental variables, statistical tests and *p*-values associated with this study are listed in Supplementary Table 1.

### Reporting summary

Further information on research design is available in the Nature Research Reporting Summary linked to this article.

## Supplementary information

Supplementary Information

Peer Review File

Description of Additional Supplementary Files

Supplementary Movie 1

Supplementary Movie 2

Supplementary Movie 3

Supplementary Movie 4

Supplementary Movie 5

Supplementary Movie 6

Supplementary Movie 7

Supplementary Movie 8

Supplementary Movie 9

Supplementary Movie 10

Supplementary Movie 11

Supplementary Movie 12

Reporting Summary

## Data Availability

The data that support the findings of this study are available from the corresponding authors upon request.
