# Supplementary material for: MT1-MMP directs force-producing proteolytic contacts that drive tumor cell invasion
Source: Nat Commun. 2019 Oct 25;10:4886. doi: 10.1038/s41467-019-12930-y (PMC6814785; doi:10.1038/s41467-019-12930-y)
Supplement: Supplementary file 1 — Supplementary Information [file 41467_2019_12930_MOESM1_ESM.pdf]

Supplementary Information

“MT1-MMP directs force-producing proteolytic contacts that drive tumor cell invasion”, Ferrari *et al*.

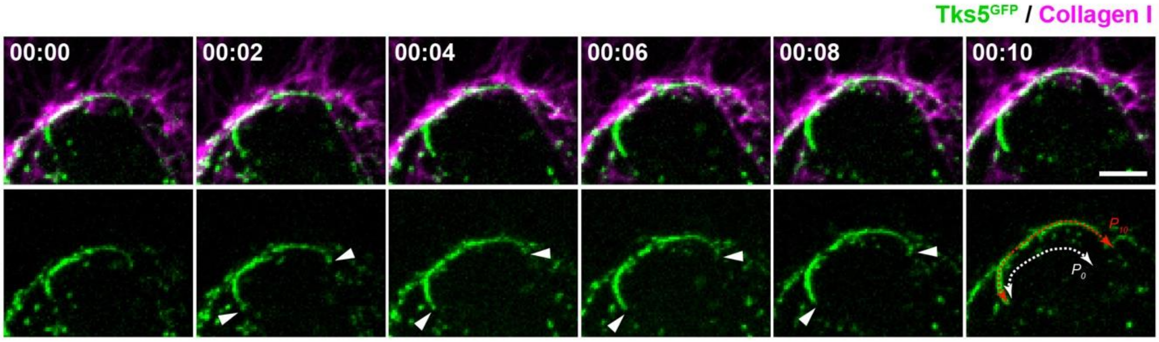

Supplementary Figure 1. Expansion of Tks5^{GFP}-positive invadopodia in association with the collagen fiber (supplementary figure to Figure 2). Gallery of non-consecutive frames (time in hr:min) of a time-lapse sequence of MDA-MB-231 cells expressing Tks5^{GFP} (green) plated on top of type I collagen (magenta). Invadopodia elongation along the associated collagen fiber is indicated by arrowheads. Invadopodia length is indicated at time 0 and after 10 min with white and red dashed lines, respectively. Scale bar, 5 µm.

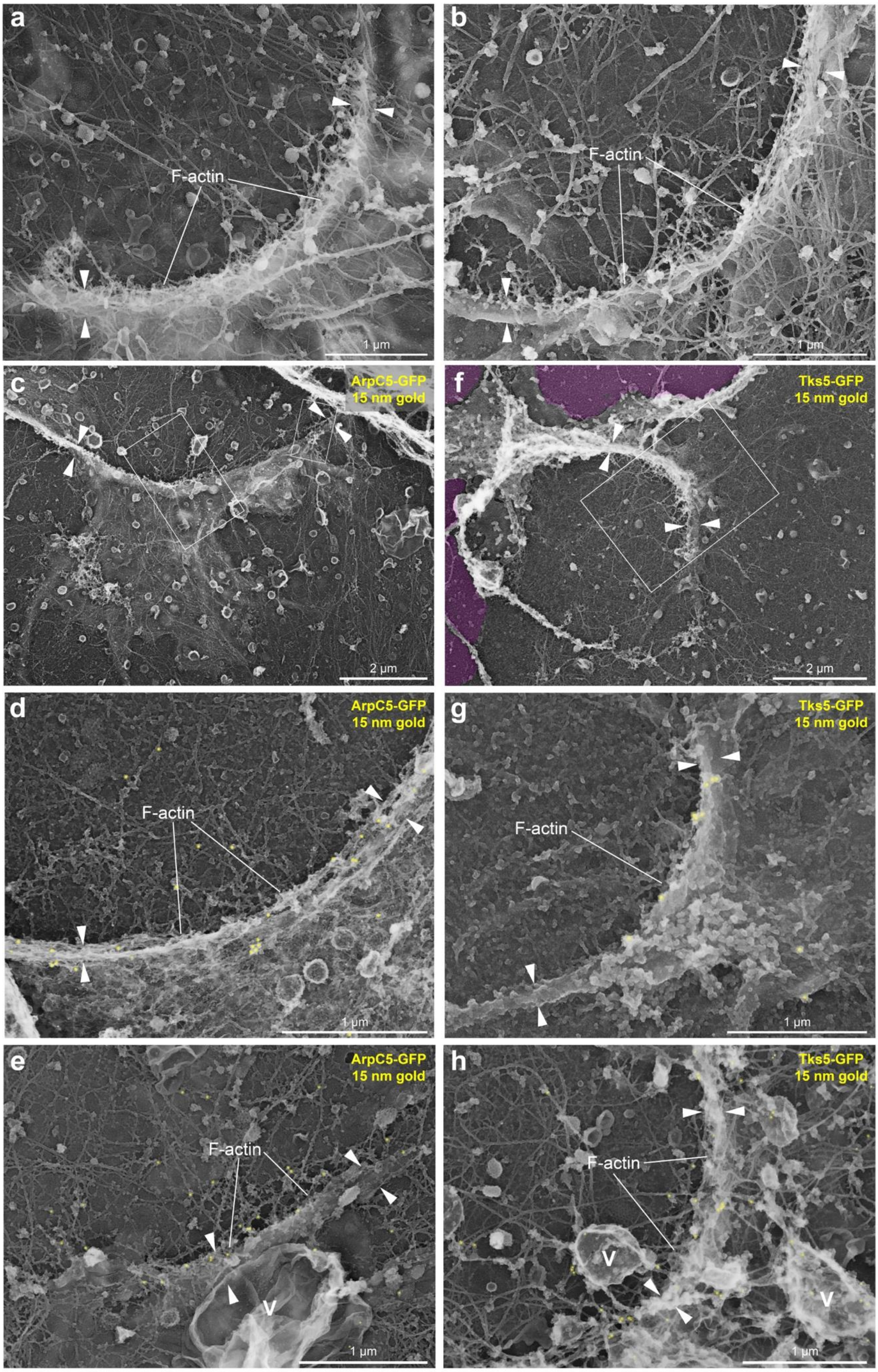
a
F-actin
1 µm
b
F-actin
1 µm
c
ArpC5-GFP
15 nm gold
2 µm
f
Tks5-GFP
15 nm gold
2 µm
d
ArpC5-GFP
15 nm gold
F-actin
1 µm
g
Tks5-GFP
15 nm gold
F-actin
1 µm
e
ArpC5-GFP
15 nm gold
F-actin
V
1 µm
h
Tks5-GFP
15 nm gold
F-actin
V
V
1 µm

Supplementary Figure 2. Invadopodia ultrastructural organization (supplementary figure to Figure 4).

(a-b) PREM images of two independent MDA-MB-231 cells plated for 60 min on a thin layer of collagen I and showing proteinaceous material in association with curvilinear collagen fibers underneath the ventral plasma membrane (indicated by arrowheads throughout the figure). Branched actin filaments along the concave side of the fiber are also visible (F-actin). Scale bars, 1 μm. **(c)** Zoom-out image of the anti-GFP immunogold PREM image shown in Figure 4e (boxed region) of MDA-MB-231 cells expressing Arp2/3 complex subunit ArpC5B^{GFP}. Scale bar, 2 μm. **(d-e)** Independent anti-GFP immunogold PREM images of MDA-MB-231 cells expressing Arp2/3 complex subunit ArpC5B^{GFP}. Immunogold beads are pseudo-colored in yellow. Branched actin networks associated with immunogold beads along the concave side of the fiber are visible (F-actin). V, large deflated vesicle in (e). Scale bar, 1 μm. **(f)** Zoom-out image of the anti-GFP immunogold PREM image shown in Figure 4g (boxed region) of MDA-MB-231 cells expressing Tks5^{GFP}. Scale bar, 2 μm. **(g-h)** Independent anti-GFP immunogold PREM images of MDA-MB-231 cells expressing Tks5^{GFP}. Immunogold particles are pseudo-colored in yellow and mostly visible along the concave side of the collagen fiber. In (g), very few actin filaments are visible along the collagen fiber suggesting a nascent invadopodia. Scale bars, 1 μm.

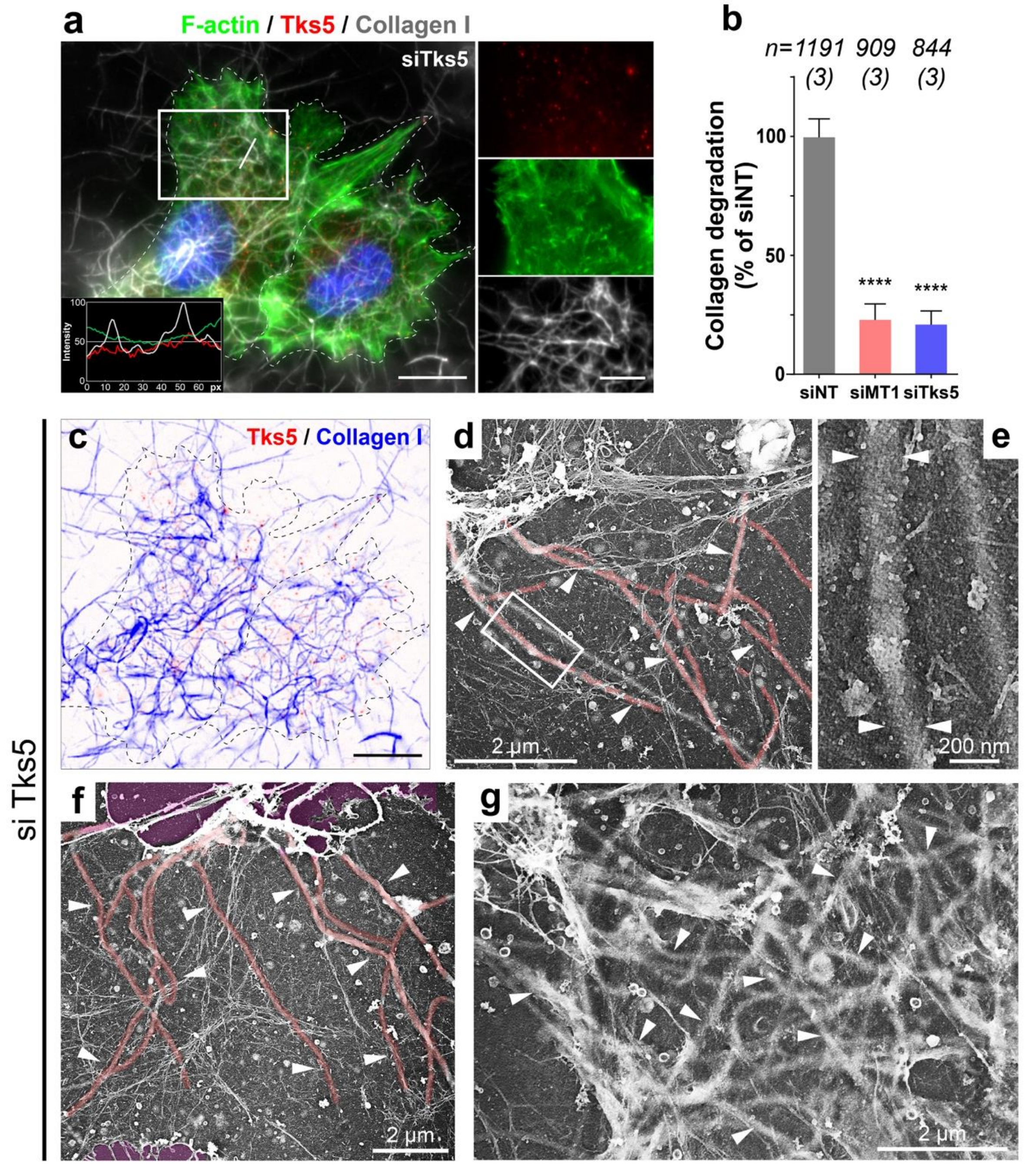

Supplementary Figure 3. Tks5 is required for the formation of collagenolytic invadopodia. (a) MDA-MB-231 cells silenced for Tks5 were plated on a layer of type I collagen (gray) and stained for F-actin (green), Tks5 (red) and DAPI (blue). Right panels show separated channels. Intensity profiles along the white line are shown in the inset. Cell contour is shown with dashed line. Scale bar, 10 µm. Zoom-in of boxed region, scale bar, 5µm. **(b)** Pericellular collagenolysis by MDA-MB-231 cells treated

with indicated siRNA measured as mean intensity of Col1-¾C signal per cell. Values for siNT-treated cells were set to 100%. n, number of cells analyzed from 3 independent experiments. Kruskal-Wallis test. **(c)** Same image as in panel (a) using inverted lookup tables (collagen fibers in blue, Tks5-positive invadopodia in red). Cell contour is shown with dashed line. Scale bar; 10 µm. **(d)** PREM images of the cytoplasmic plasma membrane surface of unroofed MDA-MB-231 cells silenced for Tks5 and plated on type I collagen. Boxed region corresponds to zoom-in image in (e). Unbent collagen fibers underneath the ventral plasma membrane are pseudo-colored in red and some are indicated with white arrowheads in panels d to g. Scale bar, 2 µm. **(e)** High magnification PREM image of the boxed region in (d). Linear collagen fibers are devoid from dense proteinaceous meshwork including actin filaments. Clathrin-coated areas are visible. Scale bars, 200 nm. **(f-g)** Independent PREM images of unroofed MDA-MB-231 cells silenced for Tks5 and plated on type I collagen. Extracellular space is pseudo-colored in purple and collagen fibers in red in (f). Scale bars, 2 µm. Error bars, SEM; ****, $P < 0.0001$.

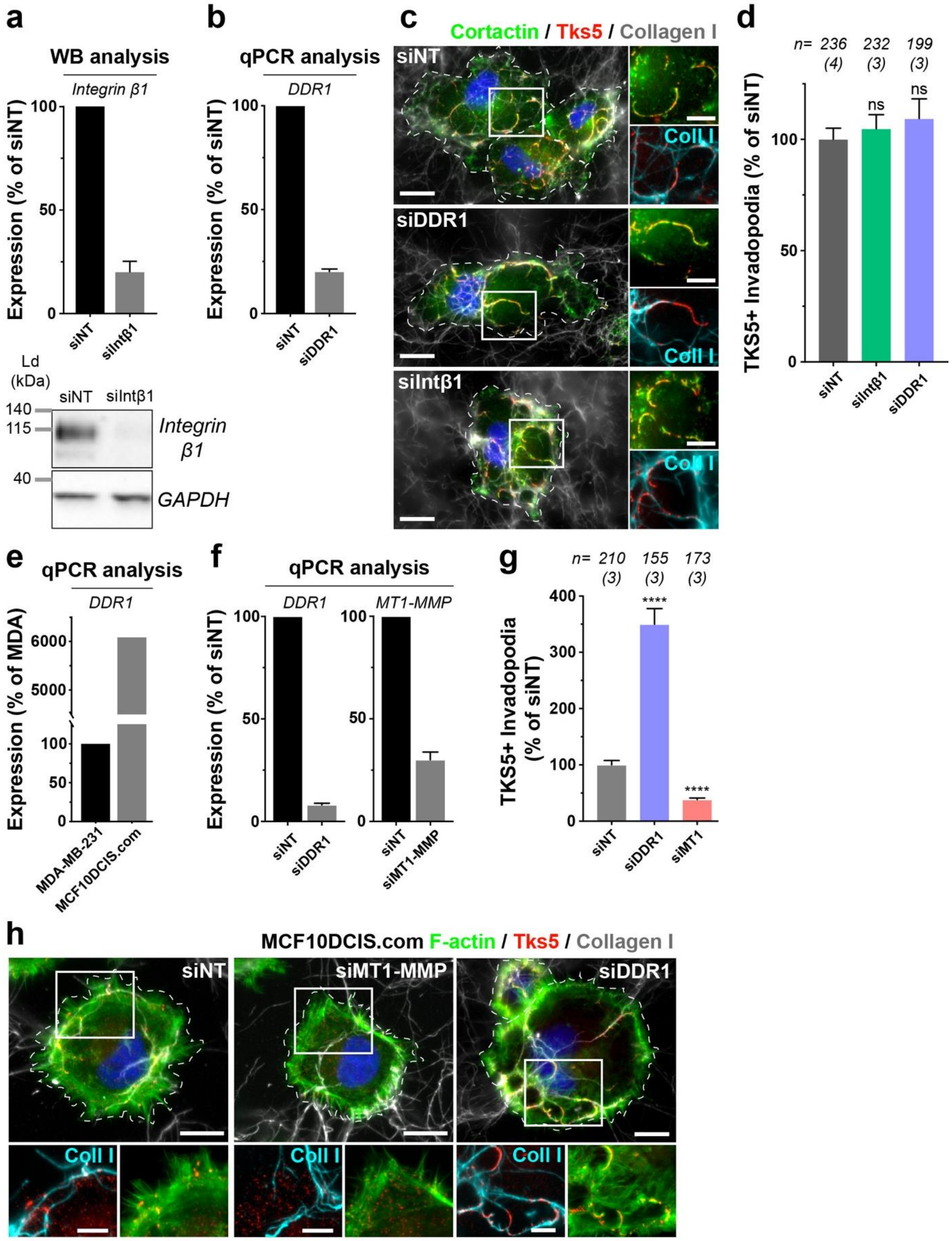

Supplementary Figure 4. DDR1 and integrin β1 collagen receptors are not required for invadopodia formation. (a) Integrin β1 expression in MDA-MB-231 cells treated with indicated siRNAs. Y-axis indicates integrin β1 expression normalized to

GAPDH expression and to mean value of siNT-treated cells (as percentage). The lower panel shows representative immunoblots of integrin β1 expression with GAPDH as loading control in indicated cell populations. **(b)** qPCR analysis of DDR1 mRNA expression in MDA-MB-231 cells treated with indicated siRNAs. Y-axis indicates DDR1 expression normalized to mean value of siNT-treated cells (as percentage). **(c)** MDA-MB-231 cells treated with indicated siRNA were plated on a layer of type I collagen (gray) and stained for cortactin (green), Tks5 (red) and DAPI (blue). Right panels are zoom-in of the boxed regions. Dashed lines, cell contour. Scale bar: 10 μm (5 μm in zoom-in insets). **(d)** Quantification of Tks5-positive invadopodia in MDA-MB-231 cells treated with indicated siRNA plated on type I collagen. Y-axis indicates ratio of the Tks5 area to total cell area normalized to mean value of siNT-treated cells (as percentage). Kruskal-Wallis test. n: number of cells; (n): number of independent experiments. **(e)** Comparative qPCR analysis of DDR1 mRNA expression in MDA-MB-231 and MCF10DCIS.com cells. Y-axis indicates DDR1 expression normalized to mean value of MDA-MB-231 cells. **(f)** qPCR analysis of DDR1 and MT1-MMP mRNA expression in MCF10DCIS.com cells treated with indicated siRNAs. Y-axis indicates DDR1 or MT1-MMP expression normalized to mean value of siNT-treated cells (as percentage). **(g)** Quantification of Tks5-positive invadopodia in MCF10DCIS.com cells treated with indicated siRNA plated on type I collagen. Y-axis indicates ratio of Tks5 area to total cell area normalized to mean value of siNT-treated cells (as percentage). Kruskal-Wallis test. n: number of cells; (n): number of independent experiments. **(h)** MCF10DCIS.com cells treated with indicated siRNAs were plated on a layer of type I collagen (gray) and stained for F-actin (green), Tks5 (red) and DAPI (blue). Lower panels are zoom-in of the boxed regions showing separated channels. Dashed lines,

cell contour. Scale bar: 10 μm (5 μm in zoom-in insets). Error bars, SEM; ****, P <0.0001; ns, non-significant.

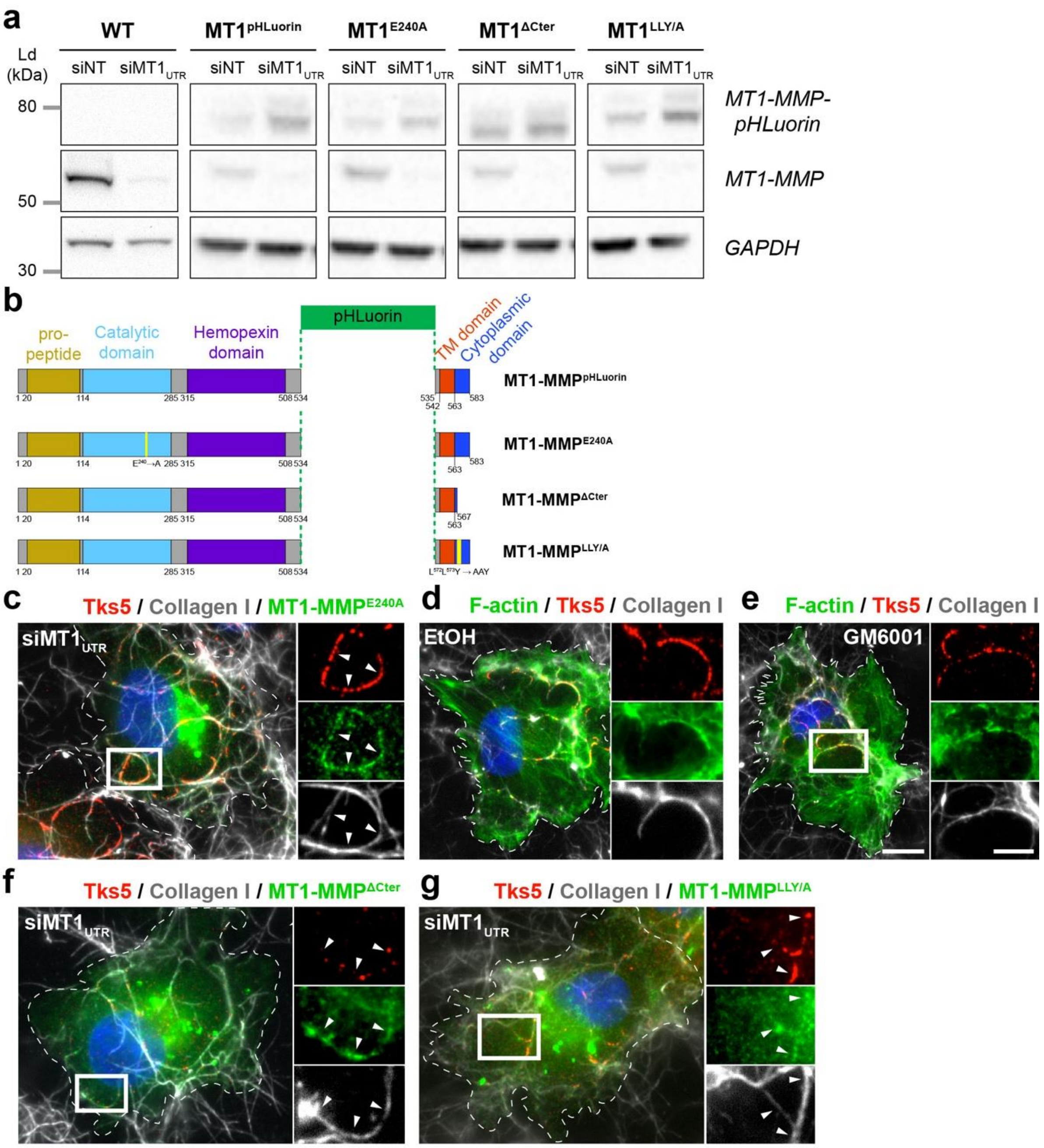

Supplementary Figure 5. Surface MT1-MMP triggers invadopodia formation (supplementary figure to Figure 5). (a) Cells were treated with siRNA against MT1-MMP 3’ and 5’ UTR sequences (siMT1$_{UTR}$, see Table S3) and transfected with indicated MT1-MMP^{pHLuorin} rescue constructs (see b). Representative immunoblotting analysis of endogenous and pHLuorin-tagged MT1-MMP expression with GAPDH as loading control. **(b)** Schematic representation of MT1-MMP^{pHLuorin} constructs used in rescue experiments. The different protein domains are colored and site of pHLuorin

insertion is depicted. Constructs with E240/A mutation in the catalytic domain, cytoplasmic tail deletion ΔCter (no C-terminus domain) and LLY/A mutation are shown with MT1-MMP amino acid numbering. **(c)** MDA-MB-231 cells treated with siMT1$_{UTR}$ siRNA and transfected with the MT1-MMP$^{E240/A}$ rescue construct were plated on a layer of collagen (grey) and stained for F-actin (green), Tks5 (red) and DAPI (blue). Right panels are zoom-in of the boxed region with separated channels. Full arrowheads point to surface MT1-MMP-pHLuorin associating with Tks5-positive invadopodia. Scale bars, 10 µm; 5 µm in zoom-in. **(d, e)** MDA-MB-231 cells mock-treated with ethanol vehicle (EtOH, panel d) or treated with GM6001 (40 µM, panel e) were plated on a layer of type I collagen (gray) and stained for F-actin (green), Tks5 (red) and DAPI (blue). Right panels are zoom-in of the boxed regions showing separated channels. Scale bars, 10 µm; 5 µm in zoom-in. **(f-g)** MDA-MB-231 cells treated with siMT1$_{UTR}$ siRNAs and transfected with the indicated (MT1-MMP$^{\Delta Cter}$ or MT1-MMP$^{LLY/A}$) rescue constructs were plated on a layer of collagen (grey) and stained for F-actin (green), Tks5 (red) and DAPI (blue). Right panels are zoom-in of the boxed region with separated channels. Full arrowheads point to surface MT1-MMP-pHLuorin associating with Tks5-positive invadopodia. Cell contour is shown with dashed lines (panels e to g). Scale bars, 10 µm; 5 µm in zoom-in.

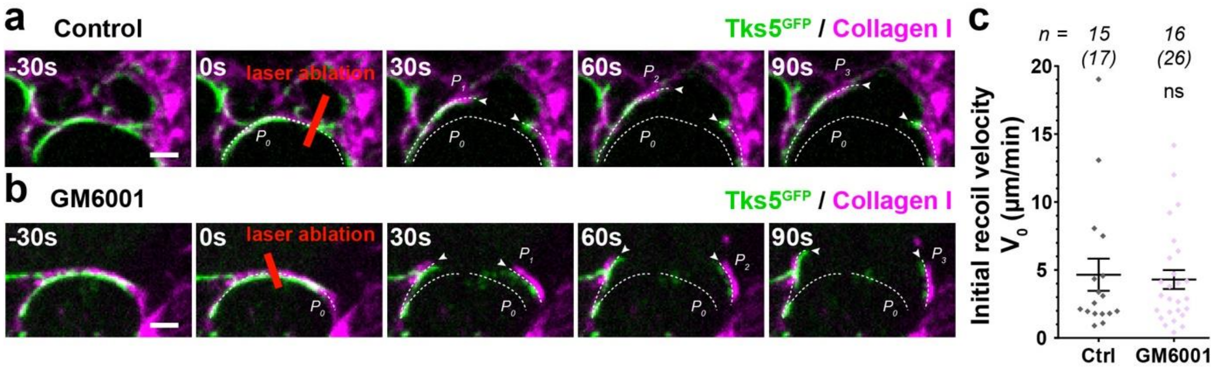

Supplementary Figure 6. Laser-induced rupture of collagen fibrils showing that invadopodia force production is independent of MT1-MMP collagenolytic activity (supplementary figure to Figure 6). (a-b) Galleries of nonconsecutive images showing Tks5^{GFP}-positive invadopodia in mock- (a) or GM6001-treated cells (b) overtime (see Movies 3 and 4). Laser ablation was performed at time 0 with the region of photo-ablation depicted in red. The position of the invadopodia tips after rupture is indicated with white arrowheads. The initial position of the invadopodia/collagen fiber ensemble (P_0) and positions after rupture (P_t) are indicated with dashed lines. Time is indicated in s. Scale bar, 2µm. **(c)** Quantification of invadopodia initial recoil velocity after laser-induced rupture ablation in Mock- or GM-treated cells. The initial recoil velocity reflects the tension stored in the structure prior rupture. Data are mean of three independent experiments. Error bars, SEM; n, number of cells analyzed, (n) number of invadopodia. Mann-Whitney test. ns, non-significant.

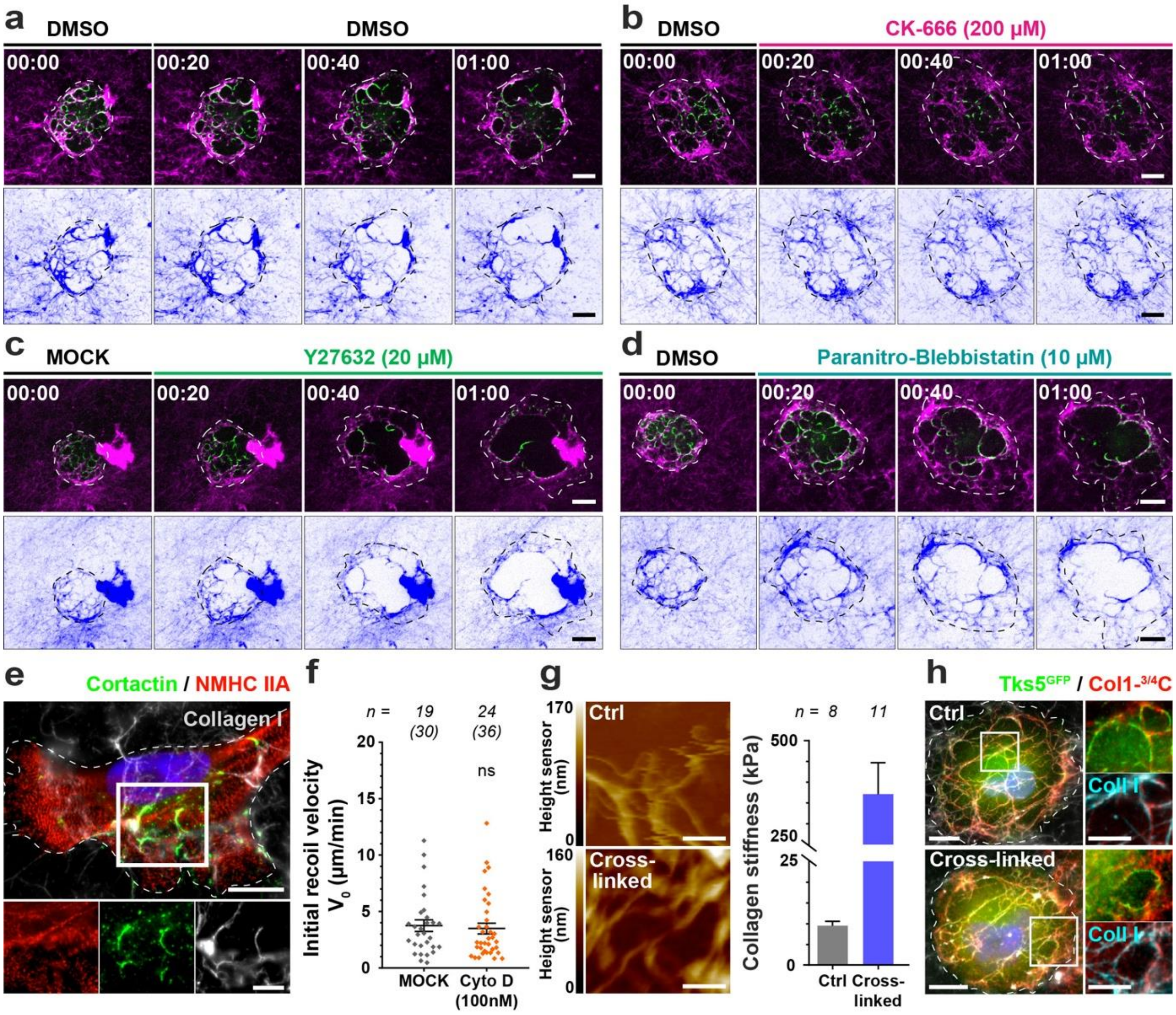

Supplementary Figure 7. Branched actin polymerization, not actomyosin activity is required for invadopodia force production (supplementary figure to Figure 7 and 8). (a-d) MDA-MB-231 cells expressing Tks5^{GFP} (green) were plated on a thin layer of type I collagen (magenta) and imaged overtime. Indicated drugs were added 15 min after starting the time-lapse. Non-consecutive frames from representative time sequences (see Movies 7 to 9) from three independent experiments are shown (time in hr:min). The collagen gel is shown in the bottom row using an inverted lookup table (collagen fibers pseudocolred blue). Scale bar; 10 µm. **(e)** MDA-MB-231 cells cultured on a type I collagen layer (gray) were stained for Cortactin (green) to label invadopodia and the heavy chain of non-muscle Myosin IIA (NMHCIIA, red) and DAPI (blue).

Bottom panels are zoom-in of the boxed region with separated channels. **(f)** Quantification of invadopodia initial recoil velocity after laser-induced rupture in Mock- or CytoD-treated cells. Data are mean of three independent experiments. Mann-Whitney test. Error bars, SEM; ns, non-significant; n, number of cells; (n) number of invadopodia. **(g)** Left panel, PeakForce Tapping atomic force microscopy (AFM) imaging of control and cross-linked (4% paraformaldehyde) collagen layers. Color-code scale of AFM height images is indicated. Scale bar, 1 µm. AFM was used to estimate the Young's modulus of control and cross-linked collagen gels (see Methods section). Data are mean of 1 experiment. Error bars, SEM; n, number of regions where stiffness was measured. **(h)** MDA-MB-231 cells expressing Tks5^{GFP} (green) were cultured on normal (top) or cross-linked (bottom) type I collagen (gray) for 60 min and stained for cleaved collagen neo-epitope (Col1-¾C, red) and DAPI (blue). Collagen proteolysis occurs in both conditions at invadopodia as shown by right panels corresponding to zoom-in of the boxed region with separated channels. The cell contour is shown with a dashed line in all panels. Scale bars, 10 µm; 5 µm in zoom-in.

Supplementary Table 1. Experimental variables measured in this study.

Variable's name (unit)	Value	SEM	P-value	n (N)	Expt	Related Figure
Collagen fiber curvature (µm^{-1})						
siNT : Inv.- fibers	0.15	0.009	-	49 (130)	3	Fig 1d
siNT : Inv.+ fibers	0.44	0.018	<0.0001	49 (127)	3	
siTks5	0.21	0.014	0.11 (ns)	41 (154)	2	
siMT1	0.21	0.012	0.05 (ns)	45 (173)	2	
XY pore cross-section (µm^2)						
siNT	3.20	0.367	-	94	2	Fig 1e
siTks5	2.29	0.482	0.0023	65	2	
siMT1	1.44	0.098	<0.0001	103	2	
Invadopodia lifetime (min)	41	1.7	-	34 (236)	2	See text
Initial recoil velocity after rupture (µm/min)	3.1	0.22	-	33 (85)	3	Fig. 2d
Distance to collagen peak of fluorescence intensity (µm)						
F-actin signal	0.129	-	-	32 (54)	2	Fig 3c
Tks5 signal	0.129	-	-	32 (54)	2	
Cortactin signal	0.0645	-	-	20 (36)	2	
Arp2/3 signal	0.0645	-	-	20 (36)	2	
Tks5+ invadopodia **(%)**						
siNT	100	4.3	-	250	3	Fig. 5d
siMT1	29.4	2.3	<0.0001	234	3	
siNT+MT1-MMP	100	6.6	-	117	4	
siMT1+MT1-MMP	109.5	8.0	0.7 (ns)	133	4	
siNT+MT1-MMP$^{\Delta C}$	100	7.0	-	109	4	
siMT1+MT1-MMP$^{\Delta C}$	43.65	4.1	<0.0001	134	4	
siNT+MT1-MMP$^{LLY/A}$	100	5.6	-	107	3	
siMT1+MT1- MMP$^{LLY/A}$	55.66	4.1	<0.0001	97	3	

EtOH	100	5.7	-	127	3	Fig 5e
GM6001	91.32	3.74	0.3 (ns)	112	3	
Rupture index (rupture events/cell/hr)						
EtOH	1.5	0.6	-	20 (42)	3	Fig. 5f
GM6001	0.4	0.1	0.007	22 (14)	3	
Invadopodia elongation rate **(µm/min)**						
MOCK	0.15	0.02	-	14 (180)	3	Fig. 6c
GM6001	0.05	0.008	<0.0001	23 (247)	3	
Invadopodia radial expansion rate **(µm/min)**						
MOCK	0.16	0.02	-	16 (29)	3	Fig. 6d
GM6001	0.05	0.005	<0.0001	19 (35)	3	
Initial recoil velocity after laser ablation (µm/min)						
MOCK	4.7	1.2	-	15 (17)	3	Fig. 6e
GM6001	4.3	0.7	0.9 (ns)	16 (26)	3	
Invadopodia elongation rate (µm/min)						
MOCK	0.12	0.008	-	60 (613)	8	Fig. 7h
Cytochalasin D (0.5 µM)	-0.15	0.01	<0.0001	17 (221)	3	
CK-666	-0.006	0.02	<0.0001	23 (190)	3	
Paranitro-blebbistatin	0.19	0.03	0.2 (ns)	22 (144)	2	
MOCK	0.14	0.03	-	18 (219)	3	
Y27632	0.18	0.01	0.8 (ns)	18 (129)	2	
Initial recoil velocity after laser ablation (µm/min)						
MOCK	3.7	0.5	-	19 (30)	3	Fig. 7i
Cytochalasin D (100 nM)	3.5	0.5	0.4 (ns)	24 (36)	3	
Invadopodia diameter growth rate (µm/min) – 3D collagen						
MOCK	0.09	0.008	-	33 (64)	3	See text
GM6001	0.03	0.006	<0.0001	34 (83)	2	
Invadopodia elongation rate (µm/min)						
MOCK	0.14	0.01	-	18 (219)	3	Fig. 8e
Cross-linked collagen (4% PFA)	0.02	0.005	<0.0001	23 (372)	3	

SEM, standard error of the mean; N, number of cells; n, number of invadopodia.

Supplementary Table 2. siRNAs used in this study.

Gene targeted	Company	Reference	Type	Targeted Sequence
**DDR1**	Dharmacon	J-003111-12-0002	Pool of 2 individuals	5'-GGGACACCCUUUGCUGGUA-3'
		J-003111-15-0002		5'-AAGAGGAGCUGACGGUUCA-3'
**ITGB1 (Integrin β1)**	Dharmacon	L-004506-00-0005	Smartpool	5'-GUGCAGAGCCUUCAAUAAA-3'
				5'-GGUAGAAAGUCGGGACAAA-3'
				5'-UGAUAGAUCCAAUGGCUUA-3'
**MMP14 (siMT1)**	Dharmacon	L-004145-00-0005	Smartpool	5'-GGAUGGACACGGAGAAUUU-3'
				5'-GGAAACAAGUACUACCGUU-3'
				5'-GGUCUCAAAUGGCAACAUA-3'
				5'-GAUCAAGGCCAAUGUUCGA-3'
**SH3PXD2A (siTKS5)**	Dharmacon	L-006657-00-0005	Smartpool	5'-ACAAUAACCUCAAAGAUGU-3'
				5'-GGACGUAGCUGUGAAGAGA-3'
				5'-CGACGGAACUCCUCCUUUA-3'
				5'-GGAUAAGUUUCCCAUUGAA-3'
**MMP14 ($siMT1_{UTR}$)**	Qiagen	SI00071169	Pool of 3 siRNAs	5'-CACAAGGACUUUGCCUCUGAA-3'
		SI05042569		5'-CCCUCAGACCUCGCUGGUAAA-3'
		SI00071190		5'-GACAGCGGUCUAGGAAUUCAA-3'
**Non-Targeting (NT)**	Dharmacon	D-001810-01-05	Individual	5'-UGGUUUACAUGUCGACUAA-3'

Supplementary Table 3. Commercial antibodies used for this study

Antigen	Company	Reference	Assay	Dilution
**Tks5**	Novus Biological	NBP1-90454	IF	1/200
			WB	1/500
**MT1-MMP**	Millipore	3328	WB	1/1000
**GAPDH**	SantaCruz Biotechnology	sc-25778	WB	1/10000
**CollI-3/4C**	ImmunoGlobe GmbH	0217-050	IF	1/100
**p34 (ARPC2B – Arp2/3 complex)**	Millipore	07-227	IF	1/50
**p16 (clone 323H3 – Arp2/3 complex)**	SYSY company	305 011	IF	1/300
**Cortactin**	Milllipore	05-180	IF	1/200
**N-WASP**	Cell signaling	4848S	IF	1/100
**GFP**	Abcam	ab13970	IF	1/2000
	Abcam	ab6556	IF	1/1000
**Paxillin**	Transduction Laboratories	610051	IF	1/1000
**Myosin II**	Covance	PRB-440P	IF	1/500
**IgG-mouse-Cy5**	Invitrogen	A31571	IF	1/500
**IgG-mouse-Cy3**	Jackson ImmunoResearch	715-165-151	IF	1/500
**IgG-mouse-A488**	Molecular Probes	A21202	IF	1/500
**IgG-mouse-Hrp**	Jackson ImmunoResearch	115-035-062	WB	1/20000
**IgG-rabbit-A488**	Molecular Probes	A11034	IF	1/200
**IgG-rabbit-Cy3**	Jackson ImmunoResearch	711-165-152	IF	1/800
**IgG-rabbit-Hrp**	Jackson ImmunoResearch	111-035-045	WB	1/10000
**IgG-rabbit-A488**	Life Technologies	A11039	IF	1/300
**Alexa Fluor 488 phalloidin**	Molecular Probes	A12379	IF	1/400
**Alexa Fluor 546 phalloidin**	Molecular Probes	A22283	IF	1/200

Supplementary Table 4. Chemical reagents used in this study.

Reagent	Company	Reference	Vehicle	Concentration
**paranitro-Blebbistatin**[a]	Optopharma	DR-N-111	DMSO	10 μM
**Y27632**	Merck	688000	Water	20 μM
**Cytochalasin D**	Merck (Sigma-aldrich)	C8273	DMSO	0.1 to 0.5 μM
**CK-666**	Merck (Sigma-aldrich)	SML0006	DMSO	200 μM
**GM 6001**	Merck (Millipore)	CC1100	Ethanol	40 μM
**Hepatocyte growth factor (HGF)**	PreproTech Inc.	100-39H	Medium	20 ng/ml

[a] Sakamoto, T., Limouze, J., Combs, C. A., Straight, A. F. & Sellers, J. R. Blebbistatin, a myosin II inhibitor, is photoinactivated by blue light. *Biochemistry* **44**, 584-588, doi:10.1021/bi0483357 (2005).

Supplementary Note 1

Model to "MT1-MMP directs force-producing proteolytic contacts that drive tumor cell invasion"

Ferrari et al.

Model hypothesis. The model that we introduce here aims at showing that protrusion forces can be generated in invadopodia/collagen-fiber ensembles in absence of solid substrate, and that these forces can lead to collagen fiber deformation. It is well established that polymerization of actin filaments can generate protrusion forces [1, 2] ; however, global force balance on a polymerizing actin meshwork requires that any protrusion force is balanced by a reaction force applied on the meshwork by its environment. This is clearly the case for adherent cellular protrusions like lamellipodia [1, 3], where a solid substrate can exert reaction forces on the actin meshwork (often mediated by focal adhesion), which in turn generates protrusive forces against the plasma membrane. In the case of 3D invadopodia that we report here, actin is polymerized along collagen fibers in absence of solid substrate to provide reaction forces, so that this classical picture does not directly apply. We argue below that shear forces in the actin meshwork, which appear when the fiber is curved, can provide locally such reaction forces to balance protrusive forces.

We consider an ECM fiber in contact with the plasma membrane of a cell. Assuming in a first approximation small deformations of the fiber, we focus on in plane deformations. We denote by L the contact length and R the mean radius of curvature of the fiber. Following experimental results, we assume that actin nucleators are recruited at the membrane along the contact line with the ECM fiber, and trigger the polymerisation of an actin meshwork inwards in the cell at speed v_p. This actin meshwork can reach a steady state thickness d because of actin depolymerisation; this thickness can be estimated as $d = v_p/k_d$, where k_d is the actin depolymerisation rate. Following classical arguments of active gel theory [4–6], which provides a coarse grained description of actin meshworks, we describe at steady state the actin layer as a fluid of viscosity η. Indeed, we are interested in time scales that are longer than the characteristic viscoelastic time scale of the actin gel (∼1-100 s.), and can therefore neglect the elastic contribution to stress [4–6]. We also neglect actin/solvent friction forces in front of shear forces in the meshwork, as was done in [7]. We show below that curvature of the fiber induces a shear stress in the actin meshwork, which in turn makes it possible for the actin meshwork to induce an outward pointing normal force on the fiber, while locally satisfying force balance. The resulting normal force on the fiber reads (per unit length):

$$\gamma \sim \frac{\eta v_p^2 a}{k_d R^2} \equiv \gamma_0 \frac{L^2}{R^2}, \qquad (1)$$

where a denote the diameter of the ECM fiber. This normal force can be interpreted as an effective 2-dimensional pressure in the plane of deformation of the fiber, which tends to push the fiber along the out pointing normal; note that it scales as $\propto 1/R^2$ and therefore increases when deformation is increased. Of note, this force vanishes for a straight fiber because shear forces vanish in absence of curvature, so that no reaction forces can be applied on the meshwork.

In turn, the deformation of an ECM fiber as an energetic cost, due to the bending elasticity of the fiber. The corresponding bending energy is given by

$$E_b \sim \frac{kTl_pL}{2R^2} \equiv e_0\frac{L^2}{R^2} \tag{2}$$

where k is the Boltzmann constant, T the temperature, and l_p the persistence length of the ECM fiber.

Last, tension τ in the fiber opposes fiber elongation and therefore deformation. For fiber bundles that are weakly cross-linked, as is expected for ECM fibres in the presence of proteolysis, the response to tension is expected to be viscoelastic ; at long time scales (larger than minutes) that are relevant to experimental observations, we therefore assume that tension is controlled by the viscous elongation of the ECM fiber, which is made possible by the relative sliding of fibrils. Such expected viscous response in the presence of proteolysis implies in particular that at rest tension can be neglected.

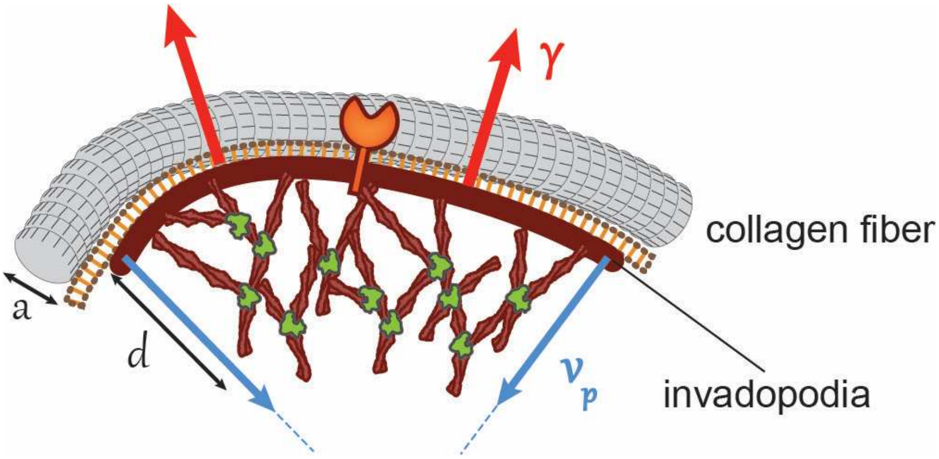

Supplementary Figure 8: Sketch of the model and main parameters.

Effective energy and polymerisation induced bending. To analyse the competi-

tion between polymerisation forces, which tend to induce deformation, and bending energy, we assume that the contact length L is fixed and analyse deformations parametrised by R only. A total effective energy E_t can then be defined from $dE_t = dE_b - \gamma dA$, where dA is the area swept by an infinitesimal shape deformation of the fiber. Denoting by $x = L/R$ the normalised curvature, this leads to :

$$E_t/e_0 = x^2 + \alpha \left(x + \sin(x) - 2\mathrm{Si}(x)\right), \tag{3}$$

where $\mathrm{Si}(x)$ is the sine integral function. The shape of the effective energy is then critically controlled by the dimensionless parameter

$$\alpha = \frac{\gamma_0 L^2}{2e_0} = \frac{\eta v_p^2 a L}{k_d k T l_p}, \tag{4}$$

as shown in Supplementary Figure 9 . For $\alpha \lesssim 10$ polymerisation forces are too weak to bend ECM fibers, and the effective energy is minimised for $x = 0$ only. For $\alpha \gtrsim 10$, the effective energy is non monotonic and displays a maximum for a critical curvature x_c and therefore a critical radius R_c. This shows that while the undeformed state ($x = 0$) is stable (we recall that in the model polymerisation forces vanish for a straight fiber, because shear forces vanish), beyond a critical deformation x_c, polymerisation forces overcome elastic forces and deform the fiber until it reaches a quasi circular shape. It is found that $R_c \sim \alpha L/10$, which shows that for large polymerisation activity (α large), any small initial curvature is sufficient to trigger large deformation to a circular shape. Importantly, in the regime of deformation where polymerisation forces overcome elastic forces, force balance on the fiber is still satisfied because of dissipative forces, such as viscous elongation of the fiber described above or viscous drag with the environment. In absence of proteolysis, fiber elongation is limited and tension increases so that only small deformations are possible, even if polymerisation forces overcome elastic bending forces.

Orders of magnitude. The critical parameter α cannot be accurately determined from experiments. Orders of magnitudes can however be inferred from the literature[6, 8]. We take the following estimates: $\eta \sim 10^4$ Pa.s ; $v_p/k_d \sim 1\mu$m ; $v_p \sim 1\mu$ms^{-1} ; $a \sim 0.1\mu$m ; $L \sim 10\mu$m ; $kTl_p \lesssim 10^{-19} J.m.$ This shows that α can reach typical values of order 10 or larger, so that polymerisation forces are sufficient to bend ECM fibers. In turn, an increase of the bending modulus leads to a decrease of α ; upon a 10 fold increase of the bending

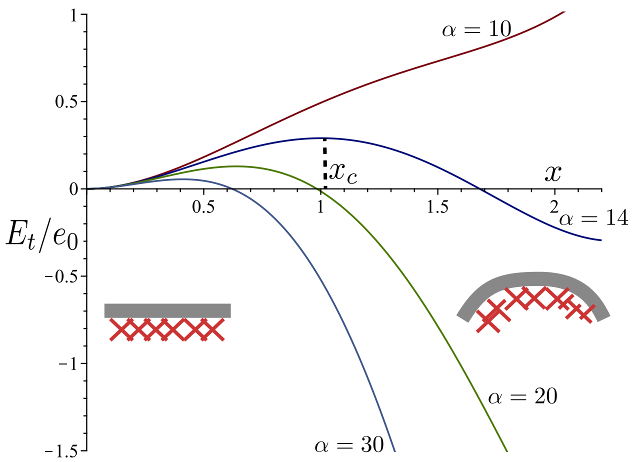

Supplementary Figure 9: Normalized effective energy as a function of the normalised curvature $x = L/R$, for different values of the critical parameter α.

modulus, α is expected to be of order 1 or smaller, so that polymerisation forces are not sufficient to bend ECM fibers.

Derivation of the polymerisation induced force. We consider radial coordinates in the plane of deformations ; the ECM fiber is delimited by the arc $r(\theta) = R$. By symmetry, we denote by $v_r \mathbf{u}_r$ the actin flow field, where $\mathbf{u}_r$ is the radial unit vector, and we neglect out of plane contributions. Boundary conditions at the ECM-membrane contact impose $v_r(R) = -v_p$; due to depolymerisation, the actin meshwork spans only the $R - d < r < R$ annular region. Force balance at the inner boundary then yields $\sigma_{rr}(R-d) = 0$. In addition we neglect pressure forces in the meshwork because it is not confined (no compression) and can exchange actin with the cytoplasm, as classically done [9–11]. Force balance then reduces to

$$0 = \partial_r \sigma_{rr} + \frac{\sigma_{rr} - \sigma_{\theta\theta}}{r}, \tag{5}$$

where the components of the viscous stress tensor are given by $\sigma_{rr} = 2\eta\partial_r v_r$ and $\sigma_{\theta\theta} = 2\eta v_r/r$. This problem can be solved explicitly for the velocity field $v_r(r)$ and yields for the stress at the ECM membrane contact:

$$\sigma_{rr}(R) = -\frac{2\eta v_p}{R}\frac{\left(1 - \frac{(R-d)^2}{R^2}\right)}{\left(1 + \frac{(R-d)^2}{R^2}\right)} \underset{d \ll R}{\sim} -\frac{2\eta v_p d}{R^2}. \tag{6}$$

This yields in turn the force per unit length exerted on the ECM fiber, as given in Eq.(1).

Last, let us comment on force balance on the actin gel, which must be satisfied. In the case of a closed circular fiber, above described normal forces exerted in reaction by the fiber on the actin gel sum to 0 and force balance is satisfied. In the case of a finite arc shaped fiber, boundary conditions are necessary and require external forces at both ends of the fiber to balance the stress $\sigma_{\theta\theta}$; this ensures global force balance on the gel. Such boundary conditions could be due to friction forces of the gel along the fiber, or local interactions with other fibers.

Supplementary References

[1] Mogilner, A. & Oster, G. Cell motility driven by actin polymerization. *Biophys J* **71**, 3030–3045 (1996).

[2] Bieling, P. *et al.* Force Feedback Controls Motor Activity and Mechanical Properties of Self-Assembling Branched Actin Networks. *Cell* **164**, 115–127 (2016).

[3] Schreiber, C. H., Stewart, M. & Duke, T. Simulation of cell motility that reproduces the force–velocity relationship. *Proceedings of the National Academy of Sciences* **107**, 9141 (2010).

[4] Kruse, K., Joanny, J. F., Julicher, F., Prost, J. & Sekimoto, K. Asters, vortices, and rotating spirals in active gels of polar filaments. *Physical Review Letters* **92**, 078101 (2004).

[5] Kruse, K., Joanny, J. F., Julicher, F., Prost, J. & Sekimoto, K. Generic theory of active polar gels: a paradigm for cytoskeletal dynamics. *European Physical Journal E* **16**, 5–16 (2005).

[6] Julicher, F., Kruse, K., Prost, J. & Joanny, J. F. Active behavior of the Cytoskeleton. *Physics Reports* **449**, 3–28 (2007).

[7] Chen, T. *et al.* Large-scale curvature sensing by directional actin flow drives cellular migration mode switching. *Nature Physics* (2019).

[8] Callan-Jones, A. C., Joanny, J.-F. & Prost, J. Viscous-Fingering-Like Instability of Cell Fragments. *Physical Review Letters* **100**, 258106 (2008).

[9] Salbreux, G., Prost, J. & Joanny, J. F. Hydrodynamics of cellular cortical flows and the formation of contractile rings. *Phys Rev Lett* **103**, 058102 (2009).

[10] Mayer, M., Depken, M., Bois, J. S., Jülicher, F. & Grill, S. W. Anisotropies in cortical tension reveal the physical basis of polarizing cortical flows. *Nature* **467**, 617–621 (2010).

[11] Ruprecht, V. *et al.* Cortical Contractility Triggers a Stochastic Switch to Fast Amoeboid Cell Motility. *Cell* **160**, 673–685 (2015).
