# Supplementary material for: MT1-MMP directs force-producing proteolytic contacts that drive tumor cell invasion
Source: Nat Commun. 2019 Oct 25;10:4886. doi: 10.1038/s41467-019-12930-y (PMC6814785; doi:10.1038/s41467-019-12930-y)
Supplement: Supplementary file 3 — Description of Additional Supplementary Files [file 41467_2019_12930_MOESM3_ESM.pdf]

Description of Additional Supplementary Files

File Name: Supplementary Movie 1
Description: **Collagenolytic invadopodia grow overtime and push collagen fibers away.** MDA-MB-231 cells expressing $Tks5^{GFP}$ (green) were plated on top of a thin type I collagen layer (magenta) and analyzed by confocal spinning-disk microscopy. Images were taken every min during 1 hr (time is in hr:min). Representative movie from three independent experiments. Asterisk: position of an unlabeled cell in the field. Scale bar, 10 µm.

File Name: Supplementary Movie 2
Description: **Collagenolytic rupture of invadopodia/collagen fiber ensemble reveals typical visco-elastic movement.** MDA-MB-231 cells expressing $Tks5^{GFP}$ (green) were plated on top of a thin type I collagen layer (magenta) and analyzed by confocal spinning-disk microscopy. Boxed regions and corresponding insets document invadopodia/collagen fiber rupture events (pointed by red arrowheads) in separated channels. Images were taken every min during 1 hr (time is in hr:min). Representative movie from three independent experiments. Scale bar, 10 µm.

File Name: Supplementary Movie 3
Description: **Inhibition of MMP proteolytic activity inhibits invadopodia expansion and elongation**. MDA-MB-231 cells expressing $Tks5^{GFP}$ (green) were treated with MMP-inhibitor GM6001 (40 µM) and plated on top of a thin type I collagen layer (magenta) before analysis by confocal spinning-disk microscopy. Images were taken every min during 1 hr (time is in hr:min). Representative movie from three independent experiments. Asterisk: position of a cell with low $Tks5^{GFP}$ expression in the field. Scale bar, 10 µm.

File Name: Supplementary Movie 4
Description: **Laser-mediated rupture of invadopodia/collagen fiber ensemble**. MDA-MB-231 cells expressing $Tks5^{GFP}$ (green) Mock- or GM6001-treated (40 µM) were plated on top of a thin type I collagen layer (magenta) and analyzed by confocal spinning-disk microscopy. Photo-ablation was performed along the region shown in red. Images were taken every 15 s (time is in hr:min:s). Representative movie from three independent experiments. Scale bar: 5 µm.

File Name: Supplementary Movie 5
Description: **Invadopodia force generation requires actin polymerization.** MDA-MB-231 cells expressing $Tks5^{GFP}$ (green) were plated on top of a thin type I collagen layer (magenta) in DMSO-treated medium and analyzed by confocal spinning-disk microscopy. Cytochalasin D (0.5 µM) was added 15 min after starting the time-lapse. Images were taken every min during 1 hr (time is in hr:min). Representative movie from three independent experiments. Scale bar, 10 µm.

File Name: Supplementary Movie 6
Description: **Arp2/3 complex function is required for invadopodia-based force generation**. MDA-MB-231 cells expressing $Tks5^{GFP}$ (green) were plated on top of a thin type I collagen layer (magenta) in DMSO-treated medium and analyzed by confocal spinning-disk microscopy. CK-666 (200 µM) was added 15 min after starting the time-lapse. Images were taken every min during 1 hr (time is in hr:min). Representative movie from three independent experiments. Scale bar, 10 µm.

File Name: Supplementary Movie 7
Description: **Invadopodia dynamics is not perturbed upon ROCK inhibition.** MDA-MB-231 cells expressing $Tks5^{GFP}$ (green) were plated on top of a thin type I collagen layer (magenta) in complete medium and analyzed by confocal spinning-disk microscopy. Y27632 (20 µM) was added 15 min after starting the time-lapse. Images were taken every min during 1 hr (time is in hr:min). Representative movie from three independent experiments. Scale bar, 10 µm.

File Name: Supplementary Movie 8
Description: **Myosin II inhibition by blebbistatin does not affect invadopodia dynamics.** MDA-MB-231 cells expressing $Tks5^{GFP}$ (green) were plated on top of a thin type I collagen layer (magenta) in DMSO-treated medium and analyzed by confocal spinning-disk microscopy. Paranitro-Blebbistatin (10 µM) was added 15 min after starting the time-lapse. Images were taken every min during 1 hr (time is in hr:min). Representative movie from three independent experiments. Scale bar, 10 µm.

File Name: Supplementary Movie 9
Description: **Tks5-positive invadopodia form as ring-like structures in 3D collagen**. Z-stack of MDA-MB-231 cells expressing $Tks5^{GFP}$ (green) embedded in a 3D fibrillar collagen-I gel (magenta) and analyzed by confocal spinning-disk microscopy. Z-steps were taken every µm as indicated and the full stack corresponds to a 15 µm-thick region in the 3D collagen gel (total width of the collagen gel is 100-150 µm). The maximal intensity z-projection corresponding to every z-steps is shown as indicated. Collagen fibers can be seen beneath and above the cell body, indicating that cells were completely embedded in the gal. Tks5-positive structures form as a ring surrounding the cell body along collagen fibers. Middle and right panels: separated channels for $Tks5^{GFP}$ and collagen signal respectively. Arrowheads point at Tks5-positive ring-like structures along collagen fibers. This stack corresponds to the 1 hr:30 min time point of Movie 2. Scale bar, 10 µm.

File Name: Supplementary Movie 10
Description: **Ring-like Tks5-positive invadopodia during 3D collagen invasion**. MDA-MB-231 cells expressing $Tks5^{GFP}$ (green) were embedded in 3D fibrillar collagen-I (magenta) and analyzed by confocal spinning-disk microscopy. Images were taken every 10 min during 15 hr (time is in hr:min). Representative movie from three independent experiments. Right panel, $Tks5^{GFP}$ signal. Arrowheads point at Tks5-positive ring-like structures along collagen fibers. Asterisk, position of cell nucleus. Scale bar, 10 µm.

File Name: Supplementary Movie 11
Description: **Abortive ring-like Tks5-positive invadopodia forming upon inhibition of MT1-MMP in 3D collagen**. MDA-MB-231 cells expressing $Tks5^{GFP}$ (green) were treated with GM6001 (40µM) and embedded in 3D fibrillar collagen-I (magenta) and analyzed by confocal spinning-disk microscopy. Images were taken every 10 min during 15 hr (time is in hr:min). Representative movie from two independent experiments. Right panel, $Tks5^{GFP}$ signal. Arrowheads point at Tks5-positive ring-like structures along collagen fibers. Asterisk, position of cell nucleus. Scale bar, 10 µm.

File Name: Supplementary Movie 12
Description: **Collagen cross-linking impairs invadopodia expansion.** MDA-MB-231 cells expressing $Tks5^{GFP}$ (green) were plated on top of a chemically (4% PFA) cross-linked type I collagen layer (magenta) and analyzed by confocal spinning-disk microscopy. Images were taken every min during 1 hr (time is in hr:min). Representative movie from three independent experiments. Asterisk: position of a cell with low $Tks5^{GFP}$ expression in the field. Scale bar, 10 µm.
