# Supplementary material for: MT1-MMP directs force-producing proteolytic contacts that drive tumor cell invasion
Source: Nat Commun. 2019 Oct 25;10:4886. doi: 10.1038/s41467-019-12930-y (PMC6814785; doi:10.1038/s41467-019-12930-y)
Supplement: Supplementary file 2 — Peer Review File [file 41467_2019_12930_MOESM2_ESM.pdf]

Reviewers' comments:

Reviewer #1 (Remarks to the Author):

In this manuscript, Ferrari and colleagues investigate the interactions of invadopodial protrusions with fibrillar type 1 collagen substrates. Extension of invadopodia is studied for cells invading into 3D gels and cultured on collagen coated substrates. The key findings reported by the authors are that: formation of invadopodia is triggered by MT1-MMP, an Arp2/3 branched actin network is associated with the concave side of curved invadopodia, and Tks5 driven actin polymerization produces forces, which along with fiber cleavage, drive invadopodial expansion.

There has been a great recent interest in invadopodial protrusions, which are thought to be critical in driving cancer invasion. This work adds our current understanding of invadopodial protrusions. In particular, the authors present some interesting data on the structure of actin networks within the invadopodia, Tks5-driven expansion of matrix pores, and the dynamics of invadopodial-collagen interactions. However, there are some significant issues with the manuscript in its current form: the advance of this work over the extensive previous work on invadopodia is not made clear and some of the conclusions are overstated, evidence for the claimed actin network architecture underlying the invadopodia needs to be strengthened, all of the studies need to be conducted in 3D collagen gels if they are to be connected to the context of migration as indicated in Figure 4, and the value of the proposed model is unclear. These issues need to be addressed in a rigorous manner.

Major Comments:
1. Conclusions overstated and advance over previous work is unclear: The coverage of previous work in the invadopodia field is inadequate, and key ideas that have already been established need to be acknowledged and discussed. For example, a number of previous papers have already shown that invadopodia generate force. For example, Qi, et al., (Journal Cell Science, 2016) show that “a strong interaction of talin2 with integrins is required to generate traction, which in turn drives invadopodium-mediated matrix degradation, which is key to cancer cell invasion”. The role of invadopodia in generating force to extend invadopodial protrusions and widen pores in the matrix, independent of their role in degradation, was also recently shown (Wisdom, et al., Nature Communications, 2018). Both of these manuscripts need to be cited and discussed. Further, the role of MT1-MMP in driving invadopodial protrusions is well established. In fact, the lead author published a review article in the journal of cell science entitled “Matrix invasion by tumour cells: a focus on MT1-MMP trafficking to invadopodia” back in 2009 in the Journal of Cell Science. Knockdown of MT1-MMP has been found to diminish invasion of cancer cells on collagen matrices (Sabeh, et al., JCB, 2004) and the authors recently revealed molecular details related to digestion of matrix by MT1-MMP that enables pore enlargement (Infante, et al., Nature Comm. 2018). While certainly the authors are revealing new molecular details on invadopodia, I cannot see how the authors have found “a new paradigm”, as is claimed in the abstract. Or, for example, the comment, “collectively, these data identified MT1-MMP as the long sought cell surface receptor required for invadopodia formation …” seems vastly overstated. Similarly overstated claims are made throughout the manuscript. The authors need to more comprehensively cite and discuss previous related literature, carefully and precisely describe the advance of this work relative to previous works, and should refrain from making such bold claims of novelty.

2. Evidence for the claimed actin network architecture underlying the invadopodia needs to be strengthened. I found figure 1, in which the authors describe the organization of collagenolytic invadopodia very difficult to follow, and the key conclusions appear to based on single zoomed-in TEM images. Some questions and suggestions:
-For figure A, it would be helpful to have a less zoomed in image of the MDA-MB-231s to see what the invadopodia protrusions look like with respect to the collagen microenvironment.
-For c – g – how do the authors know what is intracellular and extracellular? How are the authors identifying actin rather than other intracellular filamants, and collagen fibers?
-d: a zoomed in image of the actin filament architecture would be helpful.

-Zoomed out TEM images for e and f would help to get a sense of non-specific labeling, and general organization of Arp2/3 and Tks5 within the cell (showing Tks5 labeling matches with fl. Images for example).
-These conclusions in this figure are largely based on single images – is there a gallery of supporting images, or some kind of quantitative analysis that can be generated to support these conclusions?
-In g, the authors argue that collagen fibers are straight in the siTks5 cells – can this be supported with quantitative analysis?
-More generally, as the authors note, cell form an elongated invadopodia to migrate. Is the arp2/3 mediated branched actin structures/Tks5 rings formed at the base of the cells forming multiple invadopodial protrusions relevant to the elongated invadopodia? I would suggest that the authors repeat some subset of this analysis with an elongated invadopodia at a region further away from the cell body to show a similar architecture along the length of the invadopodia.

3. All studies should be conducted in 3D assays. The authors motivate this study by noting the limitations of studying invadopodia on quasi-3D substrates, and the main conclusions are related to the context of 3D cell migration (i.e. Fig. 4). However, some of the key mechanistic studies are conducted in 2.5 D, or cells plated on a very thin layer of collagen on a substrate (i.e. starting in line 158). These studies should be done in 3D, as it is not clear that the protrusions formed in 2.5D will be the same and whether these results would hold for the context of invadopodia mediated migration in 3D.

4. Proposed model needs to be clarified. I had a very difficult time following the model, and also understanding the contribution of the model to the manuscript. Physically, this seems like a very simple scenario. Arp2/3 mediated actin polymerization is well known to generate protrusive forces (i.e. Bieling, Cell, 2016), and in this geometry, those protrusive forces are acting on the surrounding matrix/collagen fibers. This could be modeled simply as a distributed load (from polymerization of actin) applied to a beam (collagen fiber), which would cause bending. This is at its essence an elementary mechanics text book problem. How is the model adding to our insight of this interaction?

If the authors do want to include their model though, there are some questions: why is the actin network model as a fluid? Arp2/3 branched actin networks are viscoelastic gels with an elastic modulus on the order of several kPa (Bieling, et al., Cell, 2016) and not viscous fluids. Further, as Arp2/3 branched actin networks are protrusive, it seems like they would be under compression due to resistance of the collagen fiber, whereas the authors note they are considering only shear stresses? The authors note that “This shows that while the undeformed state ($x = 0$) is stable, beyond a critical deformation xc, polymerisation forces overcome elastic forces and deform the fiber until it reaches a quasi circular shape.” Any transverse force on a beam will cause bending – its not clear to me why some threshold force or initial deformation is required?

Finally, I would suggest that the donkey cartoon in Fig. 4c, and associated description, should be removed as this is not a good or supported analogy, and is more confusing than it is helpful.

Minor Comments:
1. Thickness of collagen gels for 3D experiments should be noted.
2. Figure 2a and associated video, it is unclear that migration is in 3D. Some confocal slices to show that cells are embedded would help.
3. What are the pore sizes of the collagen gels in this experiment? This would be good to report given the work of Friedl and Wolf.
4. Supplementary Fig. 2 – Quantification of integrin b1/GAPDH should be added for panel a, and raw Western blot image should be added for DDR1 analysis (panel b)
5. Supplementary Fig. 3—significance not noted.
6. The authors note “Data not shown” in multiple places. Given that there are no restrictions on supplemental information length, data should be shown as supplemental information or referenced if published previously.

7. The authors claim of increased matrix compliance induced by invadopodia in the abstract, but they have no evidence for this. This should be removed.

Reviewer #2 (Remarks to the Author):

This study provides important new insights into mechanisms of ECM remodeling by invasive tumor cells. By culturing cells within 3D gels of fibrous collagen – an ECM that closely mimics the in vivo environment – the authors show that the cells make their way through the gel by simultaneously employing two mechanisms, both of which depend on MT1-MMP. First, cells use MT1-MMP as a collagen receptor, which upon engagement with collagen initiates Arp2/3 complex-dependent actin polymerization, whereas the force generated by polymerizing actin is used to push the collagen fibers out of the way. The resulting actin-rich structures represent invadopodia, even though they have a different geometry compared with the 2D systems. Second, acting as a protease, MT1-MMP degrades the collagen matrix. The results of the study are generally supported by high quality data and accompanied by all necessary quantifications. I believe that this paper will have a significant impact in the field. I have several relatively minor comments about the overall organization of the manuscript and some data that could be of better quality.

1. The description of Results is very dense and hard to follow without looking at Supplementary data, which is inconsistent with the idea of them being supplementary. I suggest that the paper should be reorganized into a full article format with all important information appearing in main figures and with the text flowing more logically and containing all necessary transitions and explanations. As one example, at the very beginning of the Results I was struck by the statement that "…proteolytic cleavage of collagen molecules suggest…" (lines 86-87) without any preparation that the cleavage was actually evaluated, how it was evaluated and that it indeed occurred. Only after going to the cited supplemental figure 1a, it was possible to understand how the authors arrived to this conclusion. Similar examples also occur throughout the manuscript, which makes it not an easy reading.

2. Three-color data are difficult to understand. Even though authors do provide two-color combinations, as well as linescans, it is still hard to appreciate certain points presented in such figures. For example, actin enrichments at invadopodia are not visible in any of these images. Single channels would be better. They can be shown in addition to, or even instead of some two-color combinations.

3. EM data are useful and informative, but their quality could be better. Filaments and clathrin cages in the images (especially Fig. 1d) are not as sharp as they should be, but appear to fuse partially with their surroundings. This problem most likely arises from the fact that the authors used air-drying from HMDS, which can produce acceptable images at low resolution, but not at high magnification. Fixation with osmium can also contribute to poor preservation of actin filaments, as actin is known to be destroyed by osmium fixation. This problem does not preclude publication, but I recommend the authors to strive for better quality of EM in future.

3. Figure S1d aims to show an enrichment of ArpC5-GFP in invadopodia, but seems to fail to do so. It is not only invisible in the actual three-color image, but the linescan also shows no peak at the collagen/Tks5-rich fiber.

4. Figure S5e, which shows cortactin/myosin IIA/collagen, is inconclusive regarding localization of IIA. Instead of expected fibrous and punctate distribution, IIA shows diffuse staining throughout the cell.

5. Figure S5g shows AFM data, but there is no explanation in the text what it means and why we need to see them.

6. Figure S4 legend says “(a) Gallery of non-consecutive frames…” To me, they look consecutive: 0, 2, 4, etc.

7. I would like to see a better explanation of the physical model for lay audience, like myself, in the text. I can understand the polymerizing actin pushes onto collagen fiber, but I am not sure what the authors mean by the shear stress, what its origin is, and how it is linked to the Arp2/3 complex. Even the supplementary model does not help me.

Reviewer #3 (Remarks to the Author):

While most observations regarding invadopodia were performed in two dimensional matrices until recently, the manuscript by Ferrari et al. examines the organization and dynamics of invadopodia in the more physiological model of three-dimensional collagen I derived matrix. Using advanced electron microscopy imaging methods, the authors elegantly document Arp2/3-dependent branched actin assembly at the concave side of invadopodia-like protrusions. They also validate previous findings that suggested that actin polymerization at invadopodia-like protrusions of cells plated in three-dimensional collagen matrix is a mechanism for generating force that pushes the invadopod membrane outwards and enables matrix deformation and penetration of the cell. The novelty of this manuscript relies, in my opinion, in the new demonstrated mechanism by which MT1-MMP binds and accumulates in association with collagen fibers to initiate a yet-to-be defined signaling cascade which leads to TKS5 recruitment and actin polymerization at plasma membrane-collagen fibers contact sites. Together with its known collagenolytic activity, activated MT1-MMP combines actin-polymerization generated force production with extracellular matrix degradation to facilitate tumor cell invasiveness.

Major comments:

Along the manuscript, authors should distinguish more clearly between invadopodia precursors, containing established invadopodial markers such as TKS5 and cortactin, and mature, degrading (collagenolytic) invadopodia, which degrade the extracellular collagen matrix. Authors should also try to be more accurate in distinguishing between formation (assembly) and activation (maturation) of invadopodia throughout the text and especially in the results part.

The last paragraph focuses on a theoretical model that describes the force balance in the invadopodia/collagen fiber ensemble. The authors describe the model in details in the supplementary material, and mention in the text “the model predicts that the force required to remodel less compliant ECM fibers should scale up”. The authors follow by a demonstration of an increase in the elastic modulus of the collagen by 40 fold when using a chemical cross-linking with 4% formaldehyde. I would suggest that the authors test here increasing levels of matrix stiffness and demonstrate, using developed formulas in their model, how the model can predict the exact value of increase in elastic modulus, followed by experimental validation of their predictions.

Minor comments:

Please define the abbreviations 2D (two-dimensional) and 3D (three-dimentsional) once in the abstract and again in the introduction.

Abstract: please change “is based from observations” to “is based on observations”
Introduction, line 68: please change “invading though the 3D” to “invading through the 3D”

Results section, line 117: please correct the word MCF10DCIS.com

Results section, line 140: please change “constructs” to “protein”

NCOMMS-19-11804

Reviewer #1 (Remarks to the Author):

In this manuscript, Ferrari and colleagues investigate the interactions of invadopodial protrusions with fibrillar type 1 collagen substrates. Extension of invadopodia is studied for cells invading into 3D gels and cultured on collagen coated substrates. The key findings reported by the authors are that: formation of invadopodia is triggered by MT1-MMP, an Arp2/3 branched actin network is associated with the concave side of curved invadopodia, and Tks5 driven actin polymerization produces forces, which along with fiber cleavage, drive invadopodial expansion.

There has been a great recent interest in invadopodial protrusions, which are thought to be critical in driving cancer invasion. This work adds our current understanding of invadopodial protrusions. In particular, the authors present some interesting data on the structure of actin networks within the invadopodia, Tks5-driven expansion of matrix pores, and the dynamics of invadopodial-collagen interactions. However, there are some significant issues with the manuscript in its current form: the advance of this work over the extensive previous work on invadopodia is not made clear and some of the conclusions are overstated, evidence for the claimed actin network architecture underlying the invadopodia needs to be strengthened, all of the studies need to be conducted in 3D collagen gels if they are to be connected to the context of migration as indicated in Figure 4, and the value of the proposed model is unclear. These issues need to be addressed in a rigorous manner.

R. We appreciate the reviewer's positive critical comments and detailed and valuable suggestions. Thanks to his/her suggestions, new experiments and analyses have been performed and added to the manuscript, which has been revised to improve clarity.

Major Comments:

1. Conclusions overstated and advance over previous work is unclear: The coverage of previous work in the invadopodia field is inadequate, and key ideas that have already been established need to be acknowledged and discussed. For example, a number of previous papers have already shown that invadopodia generate force.

For example, Qi, et al., (Journal Cell Science, 2016) show that "a strong interaction of talin2 with integrins is required to generate traction, which in turn drives invadopodium-mediated matrix degradation, which is key to cancer cell invasion".

R. We thank the reviewer for his/her suggestion to improve coverage of previous work. The work of Qi *et al.* (J Cell Sci 2016) on traction forces promoting invadopodia formation is now cited in the revised version. Nevertheless, major differences between this work and our study somehow limit a comparative analysis. A thin gelatin (*i.e.* denatured collagen) layer is used in the study of Qi *et al.*, while we used a native (acid-extracted) matrix construct of type I collagen fibers. Moreover, Qi and colleagues argue that

strong interaction between talin-2 and β1-integrin ['*This result suggests that a strong binding of talin2 to β-integrins is required for traction force generation*'**] is required for traction force and invadopodia formation on gelatin, while we showed that β1-integrin is not required for invadopodia formation along type I collagen fibers. In addition, we did not see any co-localization between classical focal adhesion markers (vinculin, paxillin) and Tks5-positive invadopodia structures in our experimental set-up (see the figure for the reviewer appended at the end of this rebuttal letter). Finally, the authors showed that talin-2 is responsible for the formation of large and stable focal adhesions where it mediates traction forces on the substratum, and that this process is required for invadopodia formation. Differently, we showed that invadopodia are constitutively able to generate forces based on branched actin assembly. Therefore, our data argue that mechanoactive collagenolytic invadopodia studied in the present report are able to produce forces towards the ECM, while Qi *et al.* did not directly address force production by invadopodia.**

The role of invadopodia in generating force to extend invadopodial protrusions and widen pores in the matrix, independent of their role in degradation, was also recently shown (Wisdom, et al., Nature Communications, 2018).

R. We thank the referee to point to this this interesting piece of work by Wisdom and colleagues, which adds important insights to our comprehension of cell invasion in 3D matrix scaffolds and the contribution of invasive protrusions to this process. This manuscript is now cited in the Introduction and Discussion sections of the revised manuscript. Yet, as stated by Wisdom and colleagues, their study described a process of protease-independent migration. By definition invadopodia are actin-based SCM-degradative structures (see invadopodia definition stated in three most cited reviews in the invadopodia/podosome field, *i.e.* Linder TiCB 2007, Murphy & Courtneidge Nat Rev Mol Cell Biol 2011 and Eddy *et al.* TiCB 2017). Thus, the new protease-independent mechanism described by Wisdom *et al.* cannot involved invadopodia structures as it occurs in the absence of degradation and is apparently purely based on force production by the advancing cell body and transmission to the hydrogel-based plastic environment used by the authors as a mimic of the ECM/basement membrane (see also Notbohm et al. Int Biol 2015). This experimental situation is not comparable to our work in which tumor cells are able to remodel the fibrous type I collagen matrix based on the formation of bona fide invadopodia with in-built force generation and matrix degradation activities.

Further, the role of MT1-MMP in driving invadopodial protrusions is well established. In fact, the lead author published a review article in the journal of cell science entitled "Matrix invasion by tumour cells: a focus on MT1-MMP trafficking to invadopodia" back in 2009 in the Journal of Cell Science. Knockdown of MT1-MMP has been

found to diminish invasion of cancer cells on collagen matrices (Sabeh, et al., JCB, 2004) and the authors recently revealed molecular details related to digestion of matrix by MT1-MMP that enables pore enlargement (Infante, et al., Nature Comm. 2018). While certainly the authors are revealing new molecular details on invadopodia, I cannot see how the authors have found "a new paradigm", as is claimed in the abstract.

R. We agree with the referee on the fact that MT1-MMP directly contributes to invasion in cancer cells in different matrices as it has been shown by seminal works from many labs including ours. However, we would like to emphasize that, until now, this effect was thought to directly and solely rely on the ability of MT1-MMP to degrade the matrix through its proteolytic activity and thanks to mechanisms leading to its accumulation in a subdomain of the plasma membrane in contact with the matrix (*i.e.* an invadopodium). One novelty of our work relies in the new demonstrated mechanism by which MT1-MMP, in association with collagen fibers can direct the mechanism of invadopodia formation as shown by Tks5 and F-actin recruitment based a yet-to-be defined signaling cascade. In other words, in addition to its known role as the effector of matrix proteolysis associated with invadopodia, our work reveals a new initiator role of MT1-MMP in *de novo* formation of invadopodia as shown by its requirement for the recruitment of several invadopodia markers (Tks5, cortactin, F-actin,…) in discrete elongated domains along collagen fibers. We believe that these data add substantially to the current knowledge on the mechanisms by which MT1-MMP promotes invasion. In addition, we provide evidence of how invadopodia can generate and apply forces on the collagen fibers and propose a mechanism based on actin polymerization to explain the generation of these forces. All together, we believe that these data provide the basis for a new paradigm for the role of collagenolytic invadopodia with in-built ECM proteolysis and force generation dual activities. The abstract has been reworded.

Or, for example, the comment, "collectively, these data identified MT1-MMP as the long sought cell surface receptor required for invadopodia formation …" seems vastly overstated.

R. Statement based on these data has been rephrased and toned down to focus on the fact that the presence of MT1-MMP, not its catalytic activity, is required for invadopodia assembly as documented by the specific recruitment of Tks5 invadopodia marker in association with collagen fibers as shown by siRNA interference and rescue experiments (see Figure 4 of the revised manuscript).

Similarly overstated claims are made throughout the manuscript. The authors need to more comprehensively cite and discuss previous related literature, carefully and

precisely describe the advance of this work relative to previous works, and should refrain from making such bold claims of novelty.

R. As suggested by the referee, we have toned down some statements, and rephrased others to fit with current data and previous works (see our responses to more specific comments of this referee below). More generally, we tried to improve our coverage of the previous invadopodia literature. The total number of cited references is 60 in the revised article (as compared to 48 citations in the initial submission).

2. Evidence for the claimed actin network architecture underlying the invadopodia needs to be strengthened. I found figure 1, in which the authors describe the organization of collagenolytic invadopodia very difficult to follow, and the key conclusions appear to based on single zoomed-in TEM images.

R. As suggested by Referee 1, Figure 1 (and accompanying Figure 1) has been revised to better and more comprehensively describe the invadopodia organization. Data initially described in Figure 1 are now displayed in three main figures (Figure 1, 2 & 3), including a full EM figure (Figure 3) and two accompanying figures (supplementary Figure 2 & 3).

Some questions and suggestions:

-For figure A, it would be helpful to have a less zoomed in image of the MDA-MB-231s to see what the invadopodia protrusions look like with respect to the collagen microenvironment.

R. The image in Figure 1a has been zoomed out to show collagen fibers around the cell body. Of note, collagenolytic invadopodia form along the collagen fibers and are not protrusive structures.

-For c – g – how do the authors know what is intracellular and extracellular? How are the authors identifying actin rather than other intracellular filaments, and collagen fibers?

R. For clarity, extracellular regions has been pseudo-colored in purple (see low magnification PREM image in Figure 2a and in Supplementary Figures 1f and 2f). Intracellular regions were identified based on characteristic roughness of the cytoplasmic face of the plasma membrane that is visible with the PREM method.

-d: a zoomed in image of the actin filament architecture would be helpful.

R. Actin filaments were classically identified based on their characteristic ~10 nm width, which is distinct from typical 15 nm-wide intermediate filaments. We

now provide a zoomed image of the actin and intermediate filaments with the estimation of their distinct width (see Figure 2d, inset).

-Zoomed out TEM images for e and f would help to get a sense of non-specific labeling, and general organization of Arp2/3 and Tks5 within the cell (showing Tks5 labeling matches with fl. Images for example).

R. We now provide zoomed-out images of Figure 2e and Figure 2g (see Supplementary Figure 1c and 1f, respectively), as well as additional examples of cells immuno-labelled for ArpC5B-GFP (Supplementary Figure 1de) and Tks5-GFP (Supplementary Figure 1gh). Tks5 labelling is associated with the underlying collagen fibers (for instance see low magnification images in Supplementary Figure 1gh), while Arp2/3 immuno-gold staining can also be observed in other regions, always in association with branched actin networks as expected for a component of the Arp2/3 complex (Supplementary Figure 1de).

-These conclusions in this figure are largely based on single images – is there a gallery of supporting images, or some kind of quantitative analysis that can be generated to support these conclusions?

R. We have performed several new quantitative analyses based on fluorescence images to determine the curvature of the collagen fibers in association with Tks5-positive invadopodia and invadopodia-free fibers (Figure 1d). The remodeling of the collagen fibers by cancer cells was estimated by measuring the xy cross-section of the collagen matrix pores underneath the cell body in different conditions (Figure 1e). Finally, the position of several invadopodia markers with respect to the associated collagen fiber and curvature was also analyzed (Figure 2).

-In g, the authors argue that collagen fibers are straight in the siTks5 cells – can this be supported with quantitative analysis?

R. We thank the referee for his/her suggestion. We added the analysis of collagen fiber curvature in several conditions (siNT, siMT1-MMP and siTks5) in Figure 1d. This analysis is based on fitting circles along curved collagen fibers and measuring the radius of the fitted circles (described in Methods section). This analysis revealed that a low level of curvature of the collagen fibers underneath Tks5-silenced cells, similar to that observed in invadopodia-free fibers found in contact with normal cells (see Figure 1d).

-More generally, as the authors note, cell form an elongated invadopodia to migrate. Is the arp2/3 mediated branched actin structures/Tks5 rings formed at the base of the cells forming multiple invadopodial protrusions relevant to the elongated invadopodia? I would suggest that the authors repeat some subset of this analysis

with an elongated invadopodia at a region further away from the cell body to show a similar architecture along the length of the invadopodia.

R. We would like to emphasize that in MDA-MB-231 cells invading through the 3D collagen matrix, the nucleus is generally located at the cell rear (see Infante et al. Nat Comm 2018) and invadopodia correspond to Tks5-positive, F-actin-positive structures which are observed in association with constricting collagen fibers at the level of the advancing invasive cell protrusion ahead of the nucleus (now highlighted in Figure 6a and sketched out in Figure 7b). Some invadopodia may completely surround the advancing cell body as a ring, some are smaller representing ring segments (see Figure 5a and Movie 10). We have no indication that these invadopodia present differences in composition and/or organization with respect to their position along the advancing invasive protrusion (i.e. from the nucleus to the cell leading edge). When cells are plated on a thick 2.5D type collagen layer, the advancing invasive protrusion flattens and spreads over the collagen layer surface ('frustrated invasion', see scheme in Figure 7a). Again, under these conditions, we have no indication that the different invadopodia that form in association to the underlying collagen fibers differ in composition and/or organization both at the light and electron microscopy levels.

3. All studies should be conducted in 3D assays. The authors motivate this study by noting the limitations of studying invadopodia on quasi-3D substrates, and the main conclusions are related to the context of 3D cell migration (i.e. Fig. 4). However, some of the key mechanistic studies are conducted in 2.5 D, or cells plated on a very thin layer of collagen on a substrate (i.e. starting in line 158). These studies should be done in 3D, as it is not clear that the protrusions formed in 2.5D will be the same and whether these results would hold for the context of invadopodia mediated migration in 3D.

R. As suggested we have extended our data in the 3D system to show that: i) invadopodia form along collagen fibers as ring-like structures in cancer cells invading through 3D fibrillar collagen (Figure 6a and Movie 9 and 10), and ii) pharmacological inhibition of MT1-MMP with GM6001 prevents cell invasion by counteracting invadopodia expansion (Figure 6b, Movie 11).

Regarding the mechanistic aspects (i.e. experiments using drugs to target actin polymerization or myosin-II activity), experiments were performed in the 2.5D set-up, which, contrary to the 3D collagen gel set-up, allows acute treatment/delivery of the different drugs as well as precise, quantitative and reproducible measurements of invadopodia expansion and elongation rate. For instance, we observed that several drugs - including GM6001 - have limited diffusion in the 3D collagen gel and need to be added directly to the collagen polymerization mix in addition to the culture medium, thus preventing addition at a given time point during the course of the experiment (time 0) and the observation of subsequent effects on preexisting invadopodia. Addition of the drugs such as actin polymerization poisons at the beginning of the experiment

would have prevented the formation of invadopodia making it impossible to conclude about a role of actin polymerization on force production by (non-existing) invadopodia. Along the same line, experiments addressing the effect of increased matrix stiffness obtained upon treatment of the collagen gel with paraformaldehyde would not be possible in 3D with cells already embedded in the gel.

Yet, we agree with the referee that invasion has to be investigated in 3D assays and when required, we have toned down conclusions regarding invasion based on the 2.5D experimental set-up.

4. Proposed model needs to be clarified. I had a very difficult time following the model, and also understanding the contribution of the model to the manuscript. Physically, this seems like a very simple scenario. Arp2/3 mediated actin polymerization is well known to generate protrusive forces (i.e. Bieling, Cell, 2016), and in this geometry, those protrusive forces are acting on the surrounding matrix/collagen fibers. This could be modeled simply as a distributed load (from polymerization of actin) applied to a beam (collagen fiber), which would cause bending. This is at its essence an elementary mechanics text book problem. How is the model adding to our insight of this interaction?

R. The model is now presented in more details in the main text, and its novel aspects have been clarified.

Indeed, it is well known that actin polymerization can generate protrusive forces. However, it should be kept in mind that force balance must be satisfied: when applied to a polymerizing actin meshwork, or a subpart of it, it means that the protrusive force generated by the polymerizing actin meshwork must be exactly balanced by a reaction force applied by the environment on the meshwork. This reaction force if often induced by a solid substrate, as in the case of a growing lamellipodium for adherent cells or in the in vitro set-up of Bieling et al 2016. It results from Newton's third law and qualitatively simply states that a polymerizing meshwork can generate forces and push something forward only if it can push back on something else. In the case of collagenolytic invadopodia, actin is polymerized along collagen fibers and is not in contact with any other solid substrate. It is therefore not clear where the reaction force comes from, as opposed to the classical text book situation where an interaction with a solid substrate is explicitly or implicitly assumed.

The non-trivial contribution of our model is to show that for a curved geometry of collagen fibers, a solid substrate is not needed, and the polymerizing actin meshwork can generate protrusive forces. We show that protrusive forces are balanced locally by viscous shear forces in the meshwork; in other words, polymerizing actin filaments push on the collagen fibers, and this force is balanced by the viscous friction with neighboring actin filaments. Even if relatively simple and indeed seemingly close to the textbook picture, this description of the mechanism is new to the best of our knowledge. This has been clarified in the text and Supplementary Model.

If the authors do want to include their model though, there are some questions: why is the actin network model as a fluid? Arp2/3 branched actin networks are viscoelastic gels with an elastic modulus on the order of several kPa (Bieling, et al., Cell, 2016) and not viscous fluids.

R. Indeed actin networks can be described as viscoelastic gels. In this pictured, popularized by the so called active gel theory (see *e.g*. Julicher Physics Reports 2007, Prost Nature Physics 2015), the actin cytoskeleton is elastic at short time scales and viscous at long time scales, where the cross-over time is usually of the order of 1-100 seconds. We are here looking at the dynamics of invadopodia at much longer time scales, so that the actin meshwork can be considered as viscous. This approximation is standard and has been used repeatedly in the literature (see references above).

Further, as Arp2/3 branched actin networks are protrusive, it seems like they would be under compression due to resistance of the collagen fiber, whereas the authors note they are considering only shear stresses?

R. As discussed above, force balance is key to describe protrusion forces. In Bieling et al, because of confinement between solid walls, the stress in the polymerizing gel increases. In the case of invadopodia, the actin meshwork grows along a collagen fiber, but is not confined and therefore not under compression. Pressure forces (defined as the normal stress due to the local filament density) are thus neglected since the actin meshwork can exchange actin with the cytoplasm and thus relax density variations. This approximation has been used in the literature to describe the actin cortex (see refs 5-7 in Supplementary Model).

The authors note that "This shows that while the undeformed state (x = 0) is stable, beyond a critical deformation xc, polymerisation forces overcome elastic forces and deform the fiber until it reaches a quasi circular shape." Any transverse force on a beam will cause bending – its not clear to me why some threshold force or initial deformation is required?

R. As explained above, a protrusion force due to polymerization needs to be balanced by a reaction force, which, in the generic case of invadopodia, cannot be due to a solid substrate. We argue with our model that shear forces can be sufficient to provide such reaction force. However, shear forces are controlled by the geometry of the fiber. They vanish for straight fibers (this why a straight fiber is expected theoretically to be stable) and increase with curvature. Quantitatively we find that threshold value of curvature is needed for the protrusion force to overcome the elastic restoring force of the fiber. This has been clarified.

Finally, I would suggest that the donkey cartoon in Fig. 4c, and associated description, should be removed as this is not a good or supported analogy, and is more confusing than it is helpful.

R. As suggested, this cartoon has been deleted (see Figure 7 of the revised manuscript).

<u>Minor Comments:</u>

1. Thickness of collagen gels for 3D experiments should be noted.

R. We provide an estimation of the thickness of the collagen gel in the 3D set-up based on confocal imaging (see Methods section – lines 731-734).

2. Figure 2a and associated video, it is unclear that migration is in 3D. Some confocal slices to show that cells are embedded would help.

R. Movie 9 is a 15 µm-thick z-stack with images taken every µm showing that collagen fibers are visible beneath and above the cell, which is completely embedded in the collagen gel. Movie 9 also clearly shows that Tks5-positive invadopodia are forming as ring-like structures along constricting collagen fibers. Movie 9 corresponds to the 01:30 (hr:min) time point of the time sequence shown in Movie 10.

3. What are the pore sizes of the collagen gels in this experiment? This would be good to report given the work of Friedl and Wolf.

R. We quantified the pore size of the 3D collagen gel in a previous report and found a dimension of ~2.3 µm (see Infante *et al.* Nat Comm 2018). In this gel, we found that invasion was MT1-MMP-dependent. In addition, we hve now measured the cross-section (xy) of matrix pores in the 2.5D collagen gel (see Figure 1e).

4. Supplementary Fig. 2 – Quantification of integrin b1/GAPDH should be added for panel a, and raw Western blot image should be added for DDR1 analysis (panel b).

R. Quantification of integrin β1 levels in MDA-MB-231 cells from western blot has been added in Supplementary Figure 4 (panel a and b). Quantification of DDR1 mRNA levels by RT-qPCR in MDA-MB-231 and MCF10DCIS.com cells is shown in panel e and f.

5. Supplementary Fig. 3—significance not noted.

R. We apologize, significance has been added based on indicated statistical test (see Figure 4e and Supplementary Table S1).

6. The authors note “Data not shown” in multiple places. Given that there are no restrictions on supplemental information length, data should be shown as supplemental information or referenced if published previously.

R. There is no more ‘Data not shown’ occurrence in the paper. All data are provided in the main figures or as supplementary information.

7. The authors claim of increased matrix compliance induced by invadopodia in the abstract, but they have no evidence for this.

R. We rephrased the text.

Reviewer #2 (Remarks to the Author):

This study provides important new insights into mechanisms of ECM remodeling by invasive tumor cells. By culturing cells within 3D gels of fibrous collagen – an ECM that closely mimics the in vivo environment – the authors show that the cells make their way through the gel by simultaneously employing two mechanisms, both of which depend on MT1-MMP. First, cells use MT1-MMP as a collagen receptor, which upon engagement with collagen initiates Arp2/3 complex-dependent actin polymerization, whereas the force generated by polymerizing actin is used to push the collagen fibers out of the way. The resulting actin-rich structures represent invadopodia, even though they have a different geometry compared with the 2D systems. Second, acting as a protease, MT1-MMP degrades the collagen matrix. The results of the study are generally supported by high quality data and accompanied by all necessary quantifications. I believe that this paper will have a significant impact in the field. I have several relatively minor comments about the overall organization of the manuscript and some data that could be of better quality.

R. We thank the reviewer for his/her very positive comments and valuable suggestions.

1. The description of Results is very dense and hard to follow without looking at Supplementary data, which is inconsistent with the idea of them being supplementary. I suggest that the paper should be reorganized into a full article format with all important information appearing in main figures and with the text flowing more logically and containing all necessary transitions and explanations. As one example, at the very beginning of the Results I was struck by the statement that "…proteolytic cleavage of collagen molecules suggest…" (lines 86-87) without any preparation that the cleavage was actually evaluated, how it was evaluated and that it indeed occurred. Only after going to the cited supplemental figure 1a, it was possible to understand how the authors arrived to this conclusion. Similar examples also occur throughout the manuscript, which makes it not an easy reading.

R. We thank the referee for his/her suggestion and we have reorganized the revised manuscript to incorporate data initially presented as supplementary information into the main figures. Accordingly, the revised article displays seven main figures (instead of four main figures in the initial submission). Specifically, data corresponding to Figure 1 of the initial submission are now displayed in three main figures (Figure 1, 2 and 3). New analyses have been performed including the quantification of collagen fiber curvature and remodeling (Figure 1d and e) and the analysis of the position of several invadopodia markers with respect to the associated collagen fiber and curvature (Figure 2). In addition, new PREM images have been added in Figure 3 and associated Supplementary Figure 2 and 3. The introduction and the discussion sections have also been extended to introduce and discuss more clearly the main concepts and previous literature.

2. Three-color data are difficult to understand. Even though authors do provide two-color combinations, as well as linescans, it is still hard to appreciate certain points presented in such figures. For example, actin enrichments at invadopodia are not visible in any of these images. Single channels would be better. They can be shown in addition to, or even instead of some two-color combinations.

R. We are now providing individual channels for fluorescence images in the main figures. Actin enrichment is thus more clearly shown in the inset of Figure 1a. Additionally, we added a cmparative analysis of the distribution of several invadopodial markers based on averaged line-scans obtained from a large number of individual invadopodia (Figure 2).

3. EM data are useful and informative, but their quality could be better. Filaments and clathrin cages in the images (especially Fig. 1d) are not as sharp as they should be, but appear to fuse partially with their surroundings. This problem most likely arises from the fact that the authors used air-drying from HMDS, which can produce acceptable images at low resolution, but not at high magnification. Fixation with osmium can also contribute to poor preservation of actin filaments, as actin is known to be destroyed by osmium fixation. This problem does not preclude publication, but I recommend the authors to strive for better quality of EM in future.

R. We thank the referee for his/her suggestion. However, for our EM experiments, we developed a new procedure which enables fast and reproducible production of platinum replicas from unroofed cells for TEM analysis. While the vast majority of the structures are very well preserved, drying can be uneven for some including some clathrin-coated pits although these cases are rare and present in deep-etch samples too. However, as published previously, clathrin-coated pits have a very particular behavior in cells plated on collagen and tend to form tube-like structures on top of the membrane above the collagen fibers (Elkhatib *et al*. Science 2017) and therefore seem as if they were fusing partially with the membrane above the collagen fiber.

In addition, the experiments performed here (for instance, comparing different siRNA conditions) using this simplified procedure would have been impossible if we had used the classical deep-etching which requires several idiosyncratic steps such as the slam freezing or the etching parts with better quality but which are time consuming and at the expense of the reproducibility. We have also tested several concentrations of Osmium and use the lowest concentration 0.5% (routinely used for EM preparation) to minimize perturbations.

Lastly, some experiments presented here were performed using an immuno-gold labeling procedure which required softer fixation conditions and hence slightly decreased quality of the morphology.

3. Figure S1d aims to show an enrichment of ArpC5-GFP in invadopodia, but seems to fail to do so. It is not only invisible in the actual three-color image, but the linescan also shows no peak at the collagen/Tks5-rich fiber.

R. We now provide fluorescence image of the endogenous ARPC2 subunit of the Arp2/3 complex in association with the invadopodia marker Tks5. In addition, a line-scan and averaging analysis of several invadopodia stained for ARPC2 and cortactin is provided in the revised draft of the manuscript (see Figure 2). Additional PREM images of immuno-labelled cells for ARPC5-GFP have been added (Supplementary Figure 2cde).

4. Figure S5e, which shows cortactin/myosin IIA/collagen, is inconclusive regarding localization of IIA. Instead of expected fibrous and punctate distribution, IIA shows diffuse staining throughout the cell.

R. This image was replaced by a better one showing the typical striated actomyosin pattern and association with linear stress fibers and the absence of co-localization between cortactin and NMHCIIA in collagen-associated invadopodia (see Supplementary Figure 7e in the revised manuscript).

5. Figure S5g shows AFM data, but there is no explanation in the text what it means and why we need to see them.

R. AFM images captured in the PeakForce Tapping mode allowed for the selection of 'point of interest' to obtain the force curves for the estimation of Young's modulus of control and cross-linked collagen gels as described in the Methods section. This information is now provided in the text and the legend of Supplementary Figure 7g and in the Methods section).

6. Figure S4 legend says "(a) Gallery of non-consecutive frames…" To me, they look consecutive: 0, 2, 4, etc.

R. The time interval between consecutive images in these time sequences was one second. In the galleries (now Figure S7 of the revised manuscript) we show one every two frames, and thus we refer to non-consecutive frames.

7. I would like to see a better explanation of the physical model for lay audience, like myself, in the text. I can understand the polymerizing actin pushes onto collagen fiber, but I am not sure what the authors mean by the shear stress, what its origin is, and how it is linked to the Arp2/3 complex. Even the supplementary model does not help me.

R. The model is introduced in more details in the text and we provide a more qualitative description of the proposed mechanism. Please, see also our detailed response to Point 4 of Referee#1.

Reviewer #3 (Remarks to the Author):

While most observations regarding invadopodia were performed in two dimensional matrices until recently, the manuscript by Ferrari et al. examines the organization and dynamics of invadopodia in the more physiological model of three-dimensional collagen I derived matrix. Using advanced electron microscopy imaging methods, the authors elegantly document Arp2/3-dependent branched actin assembly at the concave side of invadopodia-like protrusions. They also validate previous findings that suggested that actin polymerization at invadopodia-like protrusions of cells plated in three-dimensional collagen matrix is a mechanism for generating force that pushes the invadopod membrane outwards and enables matrix deformation and penetration of the cell. The novelty of this manuscript relies, in my opinion, in the new demonstrated mechanism by which MT1-MMP binds and accumulates in association with collagen fibers to initiate a yet-to-be defined signaling cascade which leads to TKS5 recruitment and actin polymerization at plasma membrane-collagen fibers contact sites. Together with its known collagenolytic activity, activated MT1-MMP combines actin-polymerization generated force production with extracellular matrix degradation to facilitate tumor cell invasiveness.

R. We thank the reviewer for his/her positive comments and valuable suggestions.

Major comments:

Along the manuscript, authors should distinguish more clearly between invadopodia precursors, containing established invadopodial markers such as TKS5 and cortactin, and mature, degrading (collagenolytic) invadopodia, which degrade the extracellular collagen matrix.

Authors should also try to be more accurate in distinguishing between formation (assembly) and activation (maturation) of invadopodia throughout the text and especially in the results part.

R. In our experimental conditions, we have not been able to distinguish between invadopodia precursor and mature stages, i.e. when cells were fixed after 60 min plated on type I collagen and stained for Col1-$^{3/4}$C cleaved-collagen neoepitope and for Tks5 or cortactin invadopodial markers, most Tks5-, cortactin-positive structures were degradative. Our data that support an initiator role of MT1-MMP in the cascade leading to Tks5 recruitment and actin polymerization implies that some level of MT1-MMP must be present already in 'precursor' invadopodia and thus these structures may have some – low- level of matrix-degradation activity even at a very early stage.

The last paragraph focuses on a theoretical model that describes the force balance in the invadopodia/collagen fiber ensemble. The authors describe the model in details in the supplementary material, and mention in the text "the model predicts that the

force required to remodel less compliant ECM fibers should scale up". The authors follow by a demonstration of an increase in the elastic modulus of the collagen by 40 fold when using a chemical cross-linking with 4% formaldehyde. I would suggest that the authors test here increasing levels of matrix stiffness and demonstrate, using developed formulas in their model, how the model can predict the exact value of increase in elastic modulus, followed by experimental validation of their predictions.

R. First let us clarify the purpose of the model: it aims at putting forward a physical mechanism that explains the observation that invadopodia can bend collagen fibers, and at identifying the key players of this mechanism. It involves simplifying assumptions (on the mechanics and geometry of collagen fibers, rheology of actin meshwork…) and physical constants that in practice very difficult to measure accurately. The model is therefore not suited for precise quantitative predictions, but rather orders of magnitudes. For example, the model predicts the dependence of invadopodia dynamics on the bending modulus of collagen fibers; this is controlled by the parameter alpha (see Supplementary Model). For the reference collagen network, available orders of magnitude suggest alpha of the order of 10-100, which yields a significant deformation of collagen fibers. For an increase by 40-fold of the bending modulus of fibers, we expect that alpha is at most of order 1, and we predict that polymerization forces cannot deform collagen fibers. This has been clarified in the text.

<u>Minor comments:</u>

Please define the abbreviations 2D (two-dimensional) and 3D (three-dimentsional) once in the abstract and again in the introduction.

Abstract: please change "is based from observations" to "is based on observations" Introduction, line 68: please change "invading though the 3D" to "invading through the 3D"

Results section, line 117: please correct the word MCF10DCIS.com

Results section, line 140: please change "constructs" to "protein"

R. We thank the Referee for his/her careful reading of our manuscript. The suggested changes have been introduced. Of note, we obtained the human mammary epithelial MCF10DCIS.com cell line from Asterand and refer to the nomenclature used by Asterand and in Miller et al. J Natl Cancer Inst 2000.

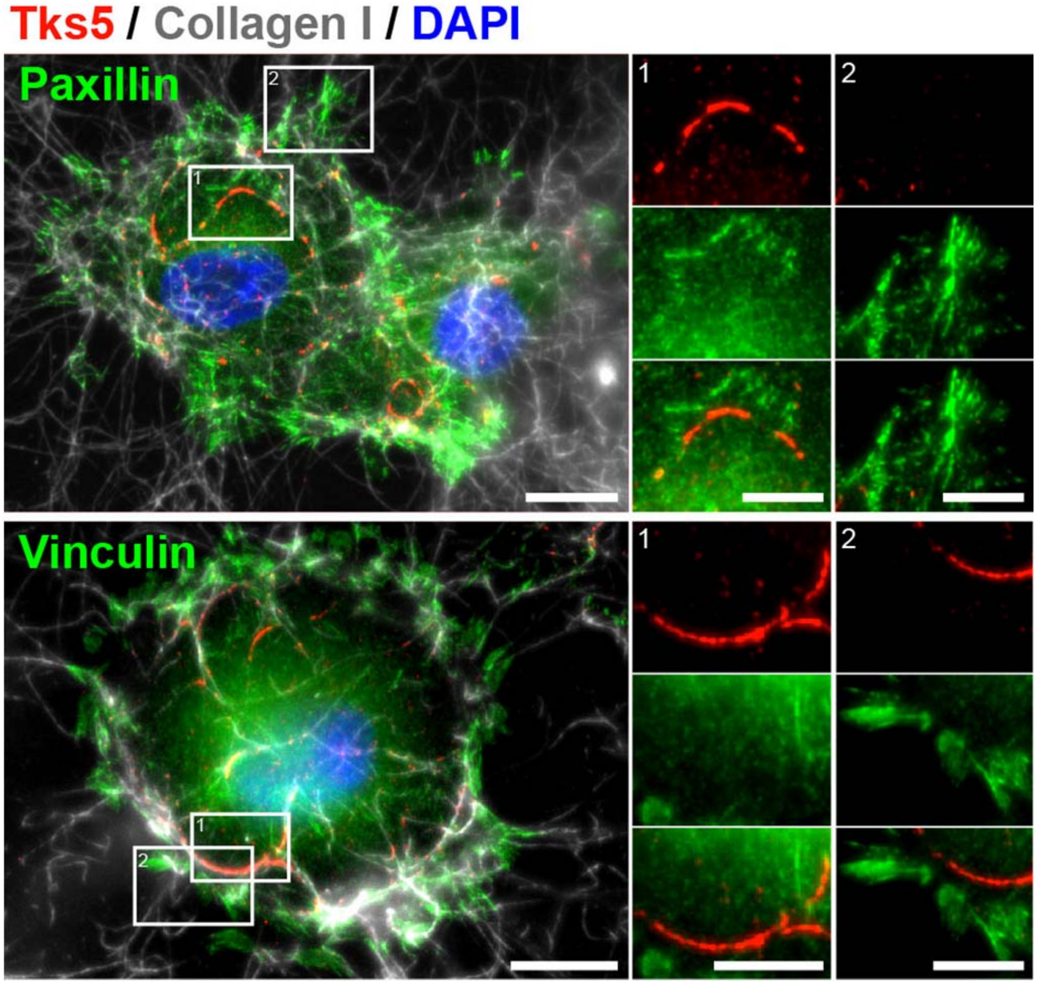

Figure for Reviewer#1 (Response to Point 1). Segregation of focal adhesions and Tks5-positive invadopodia. MDA-MB-231 cells cultured on a type I collagen layer (gray) were stained for focal adhesion proteins paxillin (upper panel) or vinculin (lower panel, green) and Tks5 (red) to label invadopodia and DAPI (blue). Right panels are zoom-in of the boxed regions with separated channels. Scale bar, 10 µm (inset, 5 µm).

REVIEWERS' COMMENTS:

Reviewer #1 (Remarks to the Author):

The authors have addressed many of the points raised in the previous round of review in their much improved revised manuscript. In my view, the key findings of this manuscript are the identification of an arp2/3 mediated network driving outward expansion of the invadopodia through force generation, and the autocrine signaling of MT1-MMP driving invadopodia assembly. There are a few remaining, mostly minor, issues that should be addressed prior to publication of this manuscript.

1. In the abstract, the phrase "… protease-independent role for MT1-MMP" is confusing given that MT1-MMP is a protease. I would suggest rewording to something like "… role for MT1-MMP in directing invadopodia assembly independent of its proteolytic activity".

2. The subtitle "MT1-MMP proteolytic activity is required for invadopodia expansion and collagen remodeling" seems too strong, given that invadopodia growth and expansion is still observed (i.e. non-zero) with GM6001 treatment. I would suggest rewording this title.

3. I am still confused with the model. I don't think the description of the actin network as viscous is accurate based on mechanical studies of Arp2/3 branched actin networks. The work of Bieling et al showed these networks to behave as elastic gels on timescales greater than several minutes. Further, the claim that collagen fibers must be curved for the invadopodia to expand still does not make sense to me.

To me, the physical picture seems simple. Actin polymerization at the membrane pushes off the existing elastic actin meshwork to extend out the membrane. If collagen fibers are in the path of this expanding invadopodia, the collagen fibers are deformed, thus applying a "reaction force" against the expanding invadopodia that puts the underlying network under compression. It is true that the polymerizing actin filaments must be pushing off something to apply a force to the collagen fibers. But it would seem that at the microscale this would be the elastic cortical actin meshwork underlying the polymerizing filament, and from a zoomed out perspective, the confining collagen matrix on the other side of the invadopodia, since the meshwork is connected like a ring around the invadopodia cross section. I would suggest that at least the authors acknowledge other possible explanations, such as that the arp2/3 network could behave like an elastic gel over the relevant timescales, in which case the collagen fibers would not need to be curved for the invadopodia to expand (as a distributed load on a straight fiber would cause it to bend).

4. As noted in my previous review, the claim that "… previous work has not demonstrated that cancer cell invadopodia are capable of producing forces" is incorrect. While the authors make a reasonable argument regarding the claims of the Qi paper, the arguments made related to the Wisdom, et al., paper are less convincing. This paper showed that invadopodial protrusions, as characterized by Tks5, generate forces in order to protrude through non-degradable matrices. The authors in their rebuttal argue that protrusions that are not actively degrading matrix cannot be invadopodia. However, the lack of degradation from the other study only seems to be because those matrices were non-degradable, and the structures extended by cells appear to be invadopodia. While this is not a major point, I do strongly feel that authors should not make claims of priority when it is not clear, especially when they aren't even needed for the paper.

5. For casual reader, I would anticipate that the figures 1 – 6 may be somewhat difficult to follow. Some schematics, similar in nature to those in Figure 7, that help convey the experimental geometry/ plane of imaging/ etc. might help clarity of the figures. This is just a suggestion; its up to the authors whether they want to do this or not.

Reviewer #2 (Remarks to the Author):

I am satisfied with the revision. The manuscript is much clearer and better presented. However, I am not fully convinced by the authors' response to my comment about EM quality. If they "developed a new procedure which enables fast and reproducible production of platinum replicas from unroofed cells for TEM analysis", as the rebuttal letter says, it is not obvious from the Methods section, which only provides a reference to the classic unroofing protocol developed by John Heuser, plus cursory description of subsequent procedures that is insufficient for those who would like to reproduce the protocol. Anyway, the current EM images are acceptable, as well as the manuscript in general. The study uncovers an important and interesting mechanism of cell migration in a 3D fibrous collagen matrix.

Reviewer #3 (Remarks to the Author):

I am satisfied with the replies and explanations of the authors to my comments. The only request that I have is to include a comment within the text regarding distinguishing (or lack of ability to distinguish) precursors vs. mature invadopodia 3D, as the authors did in their reply to my major comment 1.

NCOMMS-19-11804A

Point-by-point response to the referee's comments

We thank the three reviewers for their positive criticisms and their recommendation to publish our manuscript.

<u>Reviewer #1 (Remarks to the Author)</u>

1. In the abstract, the phrase "… protease-independent role for MT1-MMP" is confusing given that MT1-MMP is a protease. I would suggest rewording to something like "… role for MT1-MMP in directing invadopodia assembly independent of its proteolytic activity".

R. The sentence has been changed in the Abstract section according to the reviewer's suggestion (lines 31-32)

2. The subtitle "MT1-MMP proteolytic activity is required for invadopodia expansion and collagen remodeling" seems too strong, given that invadopodia growth and expansion is still observed (i.e. non-zero) with GM6001 treatment. I would suggest rewording this title.

R. The sub-title has been changed to 'MT1-MMP proteolytic activity contributes to invadopodia expansion and collagen remodeling' (line 244)

3. I am still confused with the model. I don't think the description of the actin network as viscous is accurate based on mechanical studies of Arp2/3 branched actin networks. The work of Bieling et al showed these networks to behave as elastic gels on timescales greater than several minutes. Further, the claim that collagen fibers must be curved for the invadopodia to expand still does not make sense to me.

R. Indeed the rheology of actin gels is generally found to be complex, and is classically described as viscoelastic. The viscous approximation that we take is valid in the long time regime, i-e at time scales larger than the turn-over time of actin filament, which can vary significantly in vivo or in vitro; it is generally assumed to be of the order of seconds to minutes in vivo, but is very likely to be different in in vitro experiments as described in Bieling et al. We stress that this hypothesis is standard and well referenced in the literature [see eg F. Julicher, K. Kruse, J. Prost, and J. F. Joanny. Active behavior of the cytoskeleton. Physics Reports, 449(1-3):3–28, 2007 and a vast literature by the same authors]. Importantly, the mechanism that we discuss is qualitatively independent of the specific rheology of the actin meshwork, and would not be significantly modified for a refined viscoelastic model. This has been clarified in the text.

To me, the physical picture seems simple. Actin polymerization at the membrane pushes off the existing elastic actin meshwork to extend out the membrane. If collagen fibers are in the path of this expanding invadopodia, the collagen fibers are

deformed, thus applying a "reaction force" against the expanding invadopodia that puts the underlying network under compression. It is true that the polymerizing actin filaments must be pushing off something to apply a force to the collagen fibers. But it would seem that at the microscale this would be the elastic cortical actin meshwork underlying the polymerizing filament, and from a zoomed out perspective, the confining collagen matrix on the other side of the invadopodia, since the meshwork is connected like a ring around the invadopodia cross section.
I would suggest that at least the authors acknowledge other possible explanations, such as that the arp2/3 network could behave like an elastic gel over the relevant timescales, in which case the collagen fibers would not need to be curved for the invadopodia to expand (as a distributed load on a straight fiber would cause it to bend).

R. If we interpret correctly the referee's remark, in the hypothesis of an expanding, fully-closed invadopodial F-actin ring in association with the inner rim of a ring-shape collagen matrix pore, a reaction force may come from the elastic invadopodial F-actin meshwork ring. However, fully closed invadopodia/matrix pore structures are not the only structures that were observed, partial, arc-segment shape structures were also visible and expanded over time. Thus, it is difficult to imagine that a substantial reaction force could come from the opposite side of the invadopodia. The model we developed, whereby the reaction force originates from neighbouring polymerizing actin filaments due to curvature, applies to the different types of invadopodia structures. Nevertheless, we agree that alternative mechanisms cannot be ruled out as now stated in the revised manuscript (see lines 358-366).

4. As noted in my previous review, the claim that "... previous work has not demonstrated that cancer cell invadopodia are capable of producing forces" is incorrect. While the authors make a reasonable argument regarding the claims of the Qi paper, the arguments made related to the Wisdom, et al., paper are less convincing. This paper showed that invadopodial protrusions, as characterized by Tks5, generate forces in order to protrude through non-degradable matrices. The authors in their rebuttal argue that protrusions that are not actively degrading matrix cannot be invadopodia. However, the lack of degradation from the other study only seems to be because those matrices were non-degradable, and the structures extended by cells appear to be invadopodia. While this is not a major point, I do strongly feel that authors should not make claims of priority when it is not clear, especially when they aren't even needed for the paper.

R. We removed this statement in the Discussion section (lanes 461-464) and changed our reference to the work of Wisdom et al. as suggested by this referee : 'Along this line, some pushing forces produced by Tks5-positive protrusions at the level of the advancing cell body [19,20] may trigger initial bending of constricting collagen fibers …'. (see lines 455-458)

5. For casual reader, I would anticipate that the figures 1 – 6 may be somewhat difficult to follow. Some schematics, similar in nature to those in Figure 7, that help convey the experimental geometry/ plane of imaging/ etc. might help clarity of the

figures. This is just a suggestion; its up to the authors whether they want to do this or not.

R. We thank the reviewer for his/her suggestion, however we followed the editorial recommendation not to include schematics in all the figures.

Reviewer #2 (Remarks to the Author):

I am satisfied with the revision. The manuscript is much clearer and better presented. However, I am not fully convinced by the authors' response to my comment about EM quality. If they "developed a new procedure which enables fast and reproducible production of platinum replicas from unroofed cells for TEM analysis", as the rebuttal letter says, it is not obvious from the Methods section, which only provides a reference to the classic unroofing protocol developed by John Heuser, plus cursory description of subsequent procedures that is insufficient for those who would like to reproduce the protocol. Anyway, the current EM images are acceptable, as well as the manuscript in general. The study uncovers an important and interesting mechanism of cell migration in a 3D fibrous collagen matrix.

R.: Regarding the description of the EM procedure, we now provide a more detailed version of the technique in the Methods section (lines 674-694). The unroofing method is indeed identical to the procedure developed by John Heuser, with some additional steps that are more clearly detailed in the Methods section.

Reviewer #3 (Remarks to the Author):

I am satisfied with the replies and explanations of the authors to my comments. The only request that I have is to include a comment within the text regarding distinguishing (or lack of ability to distinguish) precursors vs. mature invadopodia 3D, as the authors did in their reply to my major comment 1.

R. As suggested by this referee, we added a sentence stating that our experimental set-up did not allow to directly monitor Tks5-negative invadopodia precursors (lines 216-224).
